# ACPOA: An Adaptive Cooperative Pelican Optimization Algorithm for Global Optimization and Multilevel Thresholding Image Segmentation

**DOI:** 10.3390/biomimetics10090596

**Published:** 2025-09-06

**Authors:** YuLong Zhang, Jianfeng Wang, Xiaoyan Zhang, Bin Wang

**Affiliations:** South Korea College of Design, Hanyang University, Ansan 15588, Republic of Korea; zhangyulong0207@163.com (Y.Z.); 15306435766@163.com (J.W.); qazwsx20250811@163.com (B.W.)

**Keywords:** pelican optimization algorithm, metaheuristic algorithm, global optimization, multilevel thresholding image segmentation

## Abstract

Multi-threshold image segmentation plays an irreplaceable role in extracting discriminative structural information from complex images. It is one of the core technologies for achieving accurate target detection and regional analysis, and its segmentation accuracy directly affects the analysis quality and decision reliability in key fields such as medical imaging, remote sensing interpretation, and industrial inspection. However, most existing image segmentation algorithms suffer from slow convergence speeds and low solution accuracy. Therefore, this paper proposes an Adaptive Cooperative Pelican Optimization Algorithm (ACPOA), an improved version of the Pelican Optimization Algorithm (POA), and applies it to global optimization and multilevel threshold image segmentation tasks. ACPOA integrates three innovative strategies: the elite pool mutation strategy guides the population toward high-quality regions by constructing an elite pool composed of the three individuals with the best fitness, effectively preventing the premature loss of population diversity; the adaptive cooperative mechanism enhances search efficiency in high-dimensional spaces by dynamically allocating subgroups and dimensions and performing specialized updates to achieve division of labor and global information sharing; and the hybrid boundary handling technique adopts a probabilistic hybrid approach to deal with boundary violations, balancing exploitation, exploration, and diversity while retaining more useful search information. Comparative experiments with eight advanced algorithms on the CEC2017 and CEC2022 benchmark test suites validate the superior optimization performance of ACPOA. Moreover, when applied to multilevel threshold image segmentation tasks, ACPOA demonstrates better accuracy, stability, and efficiency in solving practical problems, providing an effective solution for complex optimization challenges.

## 1. Introduction

Image segmentation is a technique that decomposes a digital image into multiple subregions with specific features and identifies key targets. It plays a fundamental role in the fields of computer vision and pattern recognition [1,2]. With technological advancements, this technique has found significant applications in various domains such as medical image analysis, satellite remote sensing, autonomous driving, precision agriculture, and aerospace engineering. Currently, mainstream image segmentation methods can be broadly categorized into four types: threshold-based segmentation techniques, region-growing methods, clustering-based algorithms, and deep learning-based semantic segmentation networks [3,4,5].

Among these techniques, threshold segmentation has become one of the most widely adopted foundational methods in both industry and academia due to its simplicity, high computational efficiency, and reliable results [6]. Depending on specific application needs, threshold segmentation can be further divided into two forms: single-threshold segmentation, which separates foreground from background using a single threshold, and multilevel threshold segmentation, which requires determining a set of thresholds to achieve fine-grained image partitioning. Since the choice of thresholds directly affects segmentation quality and ultimately impacts the accuracy of subsequent analysis and recognition, efficiently obtaining the optimal thresholds has become a core research focus in this area. At present, Otsu’s maximum between-class variance method and Kapur’s maximum entropy method are the two most commonly used criteria for threshold selection [7,8,9].

The multilevel threshold segmentation problem is essentially a complex combinatorial optimization task. In practical applications, achieving more refined segmentation often requires setting multiple thresholds, which poses significant challenges for traditional optimization methods in terms of both computational efficiency and solution quality [10,11]. In contrast, metaheuristic algorithms inspired by the collective intelligence behaviors observed in nature have demonstrated notable advantages. These algorithms can provide high-quality solutions to multilevel threshold segmentation problems within a reasonable amount of time.

Bionics, as a bridge connecting the wisdom of natural organisms with engineering innovation, provides abundant inspiration for the design of metaheuristic algorithms. In nature, the cooperative, adaptive, and optimizing capabilities that biological populations have developed through long-term evolution serve as crucial sources for advancing algorithmic performance. For instance, ant colonies efficiently search for food paths through pheromone communication, inspiring the Ant Colony Optimization algorithm [12]; similarly, the hierarchical cooperation of gray wolves during prey hunting has given rise to the Grey Wolf Optimizer [13].These natural behaviors closely align with the core requirements of complex optimization problems, thereby offering an effective tool for their solution.

In recent years, various bio-inspired intelligent algorithms have been successfully applied in this domain. These include the Particle Swarm Optimization (PSO) algorithm inspired by the foraging behavior of bird and fish swarms [14]; the Ant Colony Optimization (ACO) algorithm based on the social foraging behavior of ants [12]; the Grey Wolf Optimizer (GWO) that mimics the social hierarchy and hunting strategy of grey wolves [13]; the Whale Optimization Algorithm (WOA), inspired by the hunting behavior of humpback whales involving search, encircling, and spiral updating phases [15]; the Dung Beetle Optimizer (DBO), which simulates behaviors such as rolling, dancing, foraging, stealing, and breeding in dung beetles [16]; the Secretary Bird Optimization Algorithm (SBOA), based on the survival behaviors of secretary birds [17]; and the Crested Porcupine Optimizer (CPO), inspired by various defense strategies of crested porcupines [18]. By simulating the intelligent behaviors of different biological populations, these algorithms have offered novel and effective approaches to solving the multilevel threshold image segmentation problem.

Bhandari et al. improved the Artificial Bee Colony (ABC) algorithm and applied it to satellite image segmentation, using Kapur, Otsu, and Tsallis functions as objective criteria to determine the optimal thresholds with ABC [19]. To enhance the real-time performance of image segmentation, Huang et al. introduced the Fruit Fly Optimization Algorithm (FOA) into Otsu-based segmentation, developing the FOA-OTSU segmentation algorithm [3]. Lin Lan proposed a novel improved African Vulture Optimization Algorithm, OLAVOA, for multilevel threshold segmentation in medical imaging [20]. Mohamed employed a hybrid approach based on the Whale Optimization Algorithm (WOA) and Moth-Flame Optimization (MFO) to determine the optimal multilevel thresholds for image segmentation tasks [21]. Abdalla introduced a WOA-based method for liver segmentation in MRI images, which extracts different clusters from abdominal images to facilitate the segmentation process [22]. Mookiah et al. proposed an Enhanced Sine Cosine Algorithm (ESCA) for determining optimal thresholds in color image segmentation [23]. Aranguren et al. developed a multilevel threshold segmentation method based on LSHADE, specifically for MRI brain imaging, and tested the proposed method using three sets of reference images [24]. Baby Resma presented a novel multilevel thresholding algorithm using the metaheuristic Krill Herd Optimization (KHO) algorithm to address the image segmentation problem. The optimal thresholds were determined by maximizing the Kapur or Otsu objective function using the KHO technique, which significantly reduced the computational time required to obtain optimal multilevel thresholds [25]. Dikshit Chauhan proposed an Artificial Electric Field Optimization (AEFO) algorithm with crossover for multilevel image segmentation [26]. Fan et al. [27] addressed the shortcomings of the traditional Moth-Flame Optimization (MFO) based Otsu image segmentation algorithm, such as low segmentation accuracy, slow convergence, and susceptibility to local optima, by proposing a fractional-order MFO-based Otsu segmentation algorithm. The approach leverages the memory and heritability properties of fractional-order calculus to control moth position updates. An adaptive fractional order is used to adjust the update mechanism based on the moth’s position, thereby improving convergence speed. The improved MFO algorithm is combined with the two-dimensional Otsu method, employing its discrete matrix to optimize the objective function [27].

As one of the most widely applied threshold-based methods for fine-grained image partitioning, multi-threshold segmentation is essentially a complex combinatorial optimization problem. With the increase in the number of thresholds (e.g., eight-level thresholding), the search space expands exponentially, rendering traditional optimization approaches (such as exhaustive search) computationally infeasible. Metaheuristic algorithms have thus become the mainstream solution, yet existing methods still face trade-offs: Particle Swarm Optimization (PSO) and Grey Wolf Optimizer (GWO) exhibit strong global exploration capability but weak local exploitation in high-dimensional threshold spaces [14]; Whale Optimization Algorithm (WOA) and Dung Beetle Optimizer (DBO) improve stability but suffer from slow convergence when handling boundary optima [15].

The Pelican Optimization Algorithm (POA) demonstrates unique advantages in multi-threshold segmentation: its simple two-phase (exploration–exploitation) framework naturally aligns with the “threshold searching–refinement” process, while its limited number of tunable parameters reduces the complexity of threshold calibration [28]. However, POA’s inherent limitations—slow convergence and low boundary accuracy—directly constrain its segmentation performance. Consequently, the combination of multi-threshold segmentation + POA emerges as a natural research focus: leveraging POA’s simplicity while addressing its drawbacks can effectively bridge the gap in efficient and accurate threshold optimization for complex images.

However, POA also has certain limitations when addressing complex optimization tasks, including slow convergence speed, low convergence accuracy, and a tendency to get trapped in local optima [5,29,30], which leads to low precision when applied to multilevel threshold image segmentation. Accordingly, many researchers have proposed improvements to POA. For example, SeyedDavoud introduced an Improved Pelican Optimization Algorithm (IPOA) that integrates three motion strategies with a predefined knowledge-sharing factor to more accurately describe the stochastic foraging behavior of pelicans, while employing a Dimension-based Hunting Learning (DHL) strategy to preserve population diversity [31]. Hao-Ming Song proposed another variant of POA that incorporates chaotic disturbance factors and basic mathematical functions: ten different chaotic disturbance factors are introduced in the exploration phase, and the best-performing scheme is then combined with six different basic functions in the exploitation phase to enhance optimization performance [32]. To address the limitations of simple strategies and susceptibility to local optima in three-dimensional UAV path planning, Guiliang Zhou developed an improved Lévy-based POA (LPOA) [33]. Furthermore, by integrating iterative chaotic mapping with refracted opposition-based learning, nonlinear inertia weight factors, Lévy flight, and an adaptive t-distribution mutation strategy, another multi-strategy improved POA (IPOA) was proposed for UAV path planning in urban environments [34]. Although these enhancements have improved POA to some extent, the algorithm still suffers from slow convergence and remains prone to premature entrapment in local optima.

To address these issues, this paper proposes an improved Adaptive Cooperative Pelican Optimization Algorithm (ACPOA). Based on the standard POA, the proposed algorithm integrates three innovative strategies: the elite pool mutation strategy guides population evolution by constructing an elite pool, thereby enhancing global exploration capability and local exploitation accuracy; the adaptive cooperative mechanism improves search efficiency in high-dimensional spaces through subgroup-based division and collaboration; and the hybrid boundary handling technique effectively preserves boundary information and improves search performance near optimal boundary solutions.

The main contributions of this paper are as follows:

(1) Proposal of the improved algorithm ACPOA: By integrating three innovative strategies into the Pelican Optimization Algorithm (POA), an Adaptive Cooperative Pelican Optimization Algorithm (ACPOA) is proposed. These strategies—namely the elite pool mutation strategy, adaptive cooperative mechanism, and hybrid boundary handling technique—effectively enhance the algorithm’s global exploration ability, local exploitation accuracy, high-dimensional search efficiency, and boundary processing capability. Together, they address the limitations of standard POA, such as premature convergence to local optima and rapid loss of population diversity in complex optimization problems;
(2) Validation of optimization performance: The performance of ACPOA is evaluated on the CEC2017 and CEC2022 benchmark test suite, in comparison with eight state-of-the-art algorithms (such as Particle Swarm Optimization (PSO), Grey Wolf Optimizer (GWO), Whale Optimization Algorithm (WOA), etc.). Statistical indicators including mean, standard deviation, and ranking are used to comprehensively demonstrate the superiority of ACPOA in solving optimization problems of various dimensions and function types, particularly in terms of convergence speed, solution accuracy, and stability;
(3) Application to multilevel threshold image segmentation: ACPOA is applied to multilevel threshold image segmentation tasks using Otsu’s method as the objective function. Segmentation is performed at 2, 4, 6, and 8 threshold levels on five benchmark images. The results, evaluated by Peak Signal-to-Noise Ratio (PSNR), Structural Similarity Index (SSIM), and Feature Similarity Index (FSIM), show that ACPOA outperforms the comparison algorithms, confirming its effectiveness in practical image processing applications.

The remainder of this paper is organized as follows: Section 2 describes the original POA and the improved ACPOA. Section 3 validates the effectiveness of ACPOA using the CEC2017 test suite and analyzes its performance. Section 4 applies the ACPOA to multi-threshold image segmentation. Section 5 concludes the study and outlines directions for future work.

## 2. Pelican Optimization Algorithm and the Proposed Methodology

### 2.1. Pelican Optimization Algorithm

#### 2.1.1. Inspiration of POA

Pelicans are large, social birds characterized by their long beaks and large throat pouches, which are used to catch and store prey. They typically weigh between 2.75 and 15 kg, stand 1.06 to 1.83 m tall, and have a wingspan ranging from 0.5 to 3 m. Pelicans primarily feed on fish, but occasionally hunt frogs, turtles, and crustaceans. They usually hunt in groups, diving from heights of 10 to 20 m to drive fish into shallow waters before capturing them. During hunting, a pelican’s beak scoops in a large volume of water, after which the excess water is expelled and the prey is swallowed [28,35]. This efficient hunting strategy demonstrates the pelican’s intelligence and cooperative behavior, which inspired the design of the Pelican Optimization Algorithm (POA). By simulating the pelican’s hunting behavior, POA achieves efficient solutions to complex optimization problems.

#### 2.1.2. Mathematical Model of POA

The POA is a population-based optimization algorithm, where each pelican represents a member of the population. In population-based algorithms, each member acts as a candidate solution, with its position in the search space corresponding to a potential solution to the optimization problem. Each population member suggests variable values for the optimization task based on its position in the search space. During the initialization phase, the positions of population members are randomly generated within the problem’s lower and upper bounds using Equation (1), which lays the foundation for the subsequent optimization process:

(1) $$\begin{array}{c} \;\;\;\;\;\;\;\;\;\;\;\;\;\;\;\;\;\;\;\;\;\;X=lb+rand\times \left(ub-lb\right) \end{array}$$

where, X represents the value of the candidate solution, ub and lb denote the upper and lower bounds of the problem, respectively, and rand is a random number in the range [0, 1].

POA simulates the behaviors and strategies of pelicans during hunting and attacking prey to update candidate solutions. This search strategy is modeled in two stages: movement toward the prey (exploration phase) and spreading wings on the water surface (exploitation phase).

(1) Exploration Phase: Movement Toward the Prey

In the first phase, pelicans identify the prey’s position and move toward that region. This behavior is modeled as the core strategy of POA, enabling the algorithm to scan and explore different areas within the search space, thereby enhancing its global exploration capability. A key feature of POA is that the prey’s position is randomly generated within the search space, which further increases the diversity and accuracy when exploring the solution space. The above concept and the pelican’s movement toward the prey are mathematically modeled by Equation (2):

(2) $$\begin{array}{c} X_{1}\left(t+1\right)=\left\{\begin{array}{c} X\left(t\right)+rand\times \left(P-I\times X\left(t\right)\right),\;\;\;\;\;\;\;\;\;\;if\;\;f\left(P\right)<f\left(X\left(t\right)\right)\\ X\left(t\right)+rand\times \left(X\left(t\right)-P\right),\;\;\;\;\;\;\;\;\;\;\;\;\;\;\;\;if\;\;f\left(P\right)\geq f\left(X\left(t\right)\right) \end{array}\right. \end{array}$$

where, $X_{1}$ denotes the pelican’s new state based on the first phase, P is the prey’s position, and *f*(∙) is the objective function representing the fitness value. I is a randomly chosen integer equal to 1 or 2, selected independently for each iteration and for each member.

(2) Exploitation Phase: Spreading Wings on the Water Surface

In the second phase, after reaching the water surface, pelicans spread their wings to drive fish toward shallow waters and then collect prey using their throat pouches. This strategy enables pelicans to capture more fish within the target area. In the Pelican Optimization Algorithm (POA), this behavior is modeled as the local search process, allowing the algorithm to converge toward better solutions within the search space. This phase enhances POA’s local search and exploitation capabilities, enabling it to find improved solutions near promising regions. Mathematically, the algorithm achieves this by examining points in the vicinity of each pelican’s position to iteratively converge toward better solutions. This hunting behavior is modeled by Equation (3), which provides theoretical support for the algorithm’s local optimization:

(3) $$\begin{array}{c} X_{2}\left(t+1\right)=X\left(t\right)+R\times \left(1-t/T\right)\times \left(2\times rand-1\right)\times X\left(t\right) \end{array}$$

where, $X_{2}$ denotes the pelican’s new state based on the second phase, R is a constant equal to 0.2, t is the current iteration number, and T is the maximum number of iterations.

During both phases, if the objective function value improves at the new position, the pelican’s position is updated; otherwise, the new position is rejected. This type of update, known as an “effective update,” prevents the algorithm from moving to non-optimal regions. This process is modeled by Equation (4):

(4) $$\begin{array}{c} X\left(t+1\right)=\left\{\begin{array}{c} X_{s}\left(t+1\right),\;\;\;\;\;\;\;\;\;\;if\;\;f\left(X_{s}\left(t+1\right)\right)<f\left(X\left(t\right)\right)\\ X\left(t\right),\;\;\;\;\;\;\;\;\;\;\;\;\;\;\;\;\;\;if\;\;f\left(X_{s}\left(t+1\right)\right)\geq f\left(X\left(t\right)\right) \end{array}\right. \end{array}$$

where, $X_{s}$ denotes the pelican’s new state in each phase, with s representing the phase and taking values 1 or 2.

### 2.2. Adaptive Cooperative Pelican Optimization Algorithm

#### 2.2.1. Elite-Pool Mutation Strategy

The standard POA algorithm in the foraging phase approaches the global optimum only through simple random perturbations. This mechanism tends to cause rapid loss of population diversity and often results in premature convergence to local optima when optimizing complex multimodal functions. To address this issue, this paper proposes an elite pool mutation strategy. The strategy constructs an elite pool consisting of the top three individuals with the best fitness. With a 10% probability, the mean of the elite individuals is used as the guiding target, while with a 90% probability, a single elite individual is randomly selected as the guiding target. The core updating formula is given by:

(5) $$\begin{array}{c} \;\;\;\;\;\;\;X_{i,j}^{1}\left(t+1\right)=\left\{\begin{array}{c} X\left(t\right)+rand\times \left(P-I\times X\left(t\right)\right),\;\;\;\;\;\;\;\;\;\;\;\;\;\;\;\;\;\;\;\;\;if\;\;f\left(P\right)<f\left(X\left(t\right)\right)\\ {Elite}_{j}\left(t\right)+rand\times \left({Elite}_{j}\left(t\right)-X_{i,j}\left(t\right)\right),\;\;\;\;\;\;if\;\;f\left(P\right)\geq f\left(X\left(t\right)\right) \end{array}\right. \end{array}$$

where the elite target Elite is generated by Equation (6):

(6) $$\begin{array}{c} Elite=\left\{\begin{array}{c} \frac{1}{3}\sum_{k=1}^{3}X_{k},\;\;\;\;if\;rand<0.1\\ X_{k},k\sim U\left\{1,\;2,\;3\right\},\;\;\;\;otherwise \end{array}\right. \end{array}$$

where, $X_{k}\;\left(k=1,\;2,\;3\right)$ denote the global best, second best, and third best solutions, respectively.

By introducing the elite pool mutation strategy, the algorithm not only retains guidance from the best individuals in the population but also maintains diversity through the random selection mechanism. This effectively enhances the local exploitation ability while avoiding premature convergence, thereby significantly improving the algorithm’s global search performance in optimizing complex multimodal functions.

#### 2.2.2. Adaptive Cooperative Mechanism

**Figure 1:**
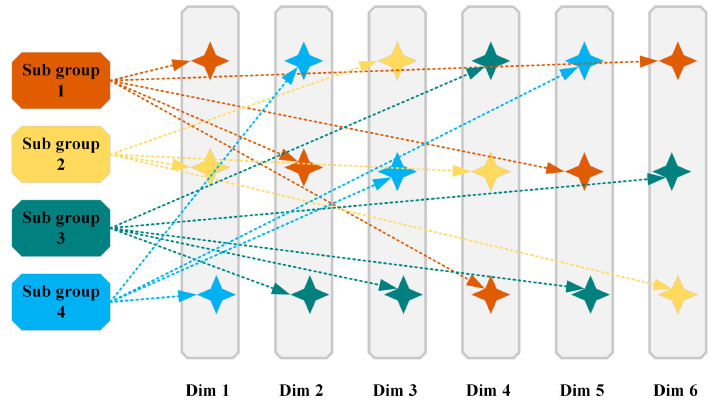
Adaptive cooperative mechanism.

The standard Pelican Optimization Algorithm (POA) employs a unified search strategy to update all dimensions of the solution vector simultaneously. This single approach struggles to adapt to the heterogeneous characteristics of different dimensions in high-dimensional optimization problems, which may lead to low search efficiency and premature convergence. To overcome this limitation, we propose an innovative adaptive cooperative mechanism that realizes specialized division of labor in the optimization process through multiple subgroups working collaboratively. This mechanism consists of two core components:

Subgroup-Dimension Allocation: Using a roulette wheel selection method, the search space is dynamically partitioned. As shown in Figure 1, the probability $p_{s,d}$ of assigning dimension d to the specialized subgroup s is defined as:

(7) $$\begin{array}{c} p_{s,d}=\frac{1}{S}\;\left(initial\;value\right),\;S=min\left(4,dim\right) \end{array}$$

here, S denotes the number of subgroups, and the probability matrix p of size S×dim guides the allocation process.

Specialized Dimension Update: Each subgroup updates only the dimensions it is responsible for, employing the standard POA two-stage update process as follows:

(8) $$\begin{array}{c} \;\;X_{i,d_{s}}^{1}\left(t+1\right)=\left\{\begin{array}{c} X_{i,d_{s}}\left(t\right)+rand\times \left(P-I\times X_{i,d_{s}}\left(t\right)\right),\;\;\;\;\;\;\;\;\;\;\;\;\;\;\;\;\;\;\;\;\;\;\;if\;\;f\left(P\right)<f\left(X\left(t\right)\right)\\ {Elite}_{d_{s}}\left(t\right)+rand\times \left({Elite}_{d_{s}}\left(t\right)-X_{i,d_{s}}\left(t\right)\right),\;\;\;\;\;\;\;\;\;\;\;\;if\;\;f\left(P\right)\geq f\left(X\left(t\right)\right) \end{array}\right. \end{array}$$

(9) $$\begin{array}{c} \;X_{i,d_{s}}^{2}\left(t+1\right)=X_{i,d_{s}}\left(t\right)+R\times \left(1-\frac{t}{T}\right)\times \left(2\times rand-1\right)\times X_{i,d_{s}}\left(t\right) \end{array}$$

where, $d_{s}$ represents the dimensions assigned to subgroup s.

This mechanism constructs a cooperative ecosystem where subgroups specialize in searching their assigned dimensions while sharing global information through the elite solution ${Elite}_{d_{s}}$. This approach effectively balances specialization and collaboration, making it particularly suitable for high-dimensional problems requiring diverse search strategies.

#### 2.2.3. Hybrid Boundary Handing

**Figure 2:**
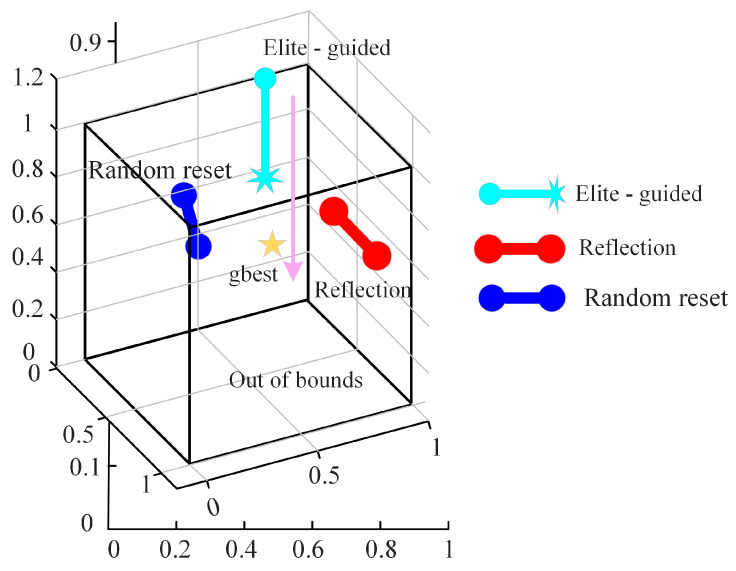
Hybrid boundary handing.

The standard POA uses a simple boundary truncation method to handle out-of-bound individuals. This approach leads to the loss of gradient information near the boundaries, resulting in low search efficiency around boundary optima. To address this issue, this paper proposes a Hybrid Boundary Handling (HBH) technique that employs a probabilistic mixture repair strategy for out-of-bound individuals. As shown in Figure 2, with a 40% probability, the individual moves toward the global best solution; with another 40% probability, a mirror reflection is applied; and with the remaining 20% probability, the position is randomly reset:

(10) $$\begin{array}{c} X_{ij}=\left\{\begin{array}{c} PZ_{j}+N\left(0,0.1\right)\times \left(ub_{j}^{\left(t\right)}-{X_{best}}_{j}\right),\;\;\;\;\;\;\;\;\;\;if\;P\leq 0.4\\ 2ub_{j}^{\left(t\right)}-X_{ij},\;\;\;\;\;\;\;\;\;\;\;\;\;\;\;\;\;\;\;\;\;\;\;\;\;\;\;\;\;\;\;\;\;\;\;\;\;\;\;\;\;\;\;\;\;\;\;if\;P\leq 0.8\\ lb_{j}^{\left(t\right)}+U\left(0,1\right)\times \left(ub_{j}^{\left(t\right)}-lb_{j}^{\left(t\right)}\right),\;\;\;\;\;\;\;\;\;\;\;\;\;\;\;if\;P>0.8 \end{array}\right. \end{array}$$

where, P is a uniformly distributed random number between 0 and 1.

This strategy offers multiple advantages by balancing exploitation (elite guidance), exploration (reflection), and diversity (random reset) in a 2:2:1 ratio. Moreover, it intelligently selects the repair method based on the degree of boundary violation and the optimization phase. Compared to the simple truncation method used in standard POA, HBH preserves more original search direction information, thereby improving boundary search efficiency.

**Figure 3:**
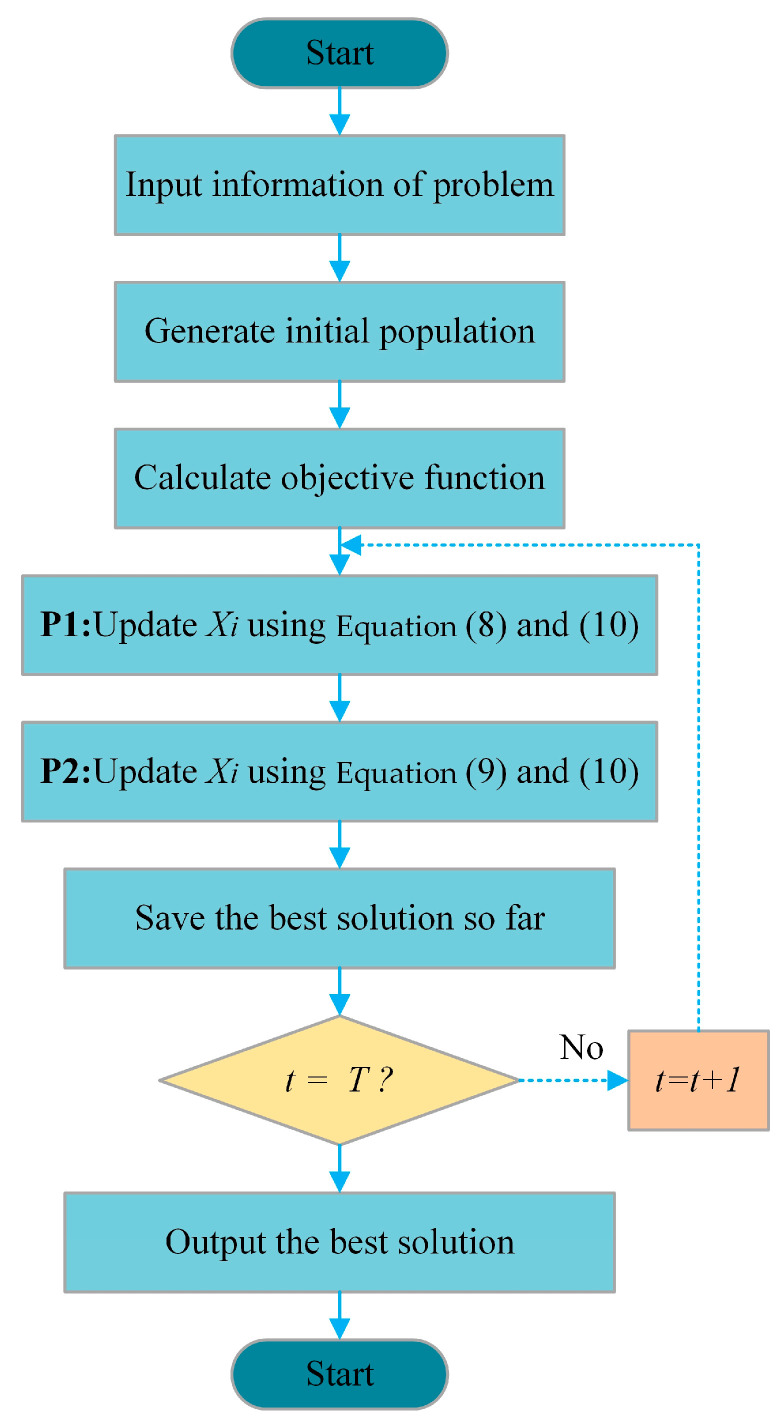
Flowchart of ACPOA.

The pseudocode for ACPOA is depicted as Algorithm 1 and the flowchart of ACPOA is shown in Figure 3.


**Algorithm 1** Pseudo-Code of ACPOA.$1:\;\mathrm{Initialize}\;\mathrm{setting}\;(\mathrm{population}\left(N\right),dim$, ub,lb$),\;\mathrm{Max}\;\mathrm{iterations}\left(T\right)$2: Initialize Elite Pool = top 3 individuals sorted by fitness. $3:\;\mathrm{for}\mathrm{each}\;\mathrm{swarm}\;i\in \left[1,K\right]$ $4:\;\;\;\;\;\;\mathrm{Find}\;S_{j}$$\;\mathrm{swarm}\;\mathrm{members}:\;\mathrm{Let}\;d_{s}$$\mathrm{be}\;\mathrm{the}\;\mathrm{set}\;\mathrm{of}\;\mathrm{corresponding}\;\mathrm{dimensions}\;\mathrm{of}\;S_{j}$$:d_{s}\subseteq |Swarm_{table\left(j,d_{s}\right)}=1$ 5:      for t = 1:T 6:          Generate the position of the prey at random 7:         fori = 1:N 8:              ***Phase 1: Moving towards prey (exploration phase)*** 9:              Calculate new status of Pelican using Equation (8) 10:              Update the population member of Pelican using Equation (4) 11:              Boundary handing using Equation (10) 12:             ***Phase 2: Winging on the water surface (exploitation phase)*** 13:             Calculate new status of Pelican using Equation (9) 14:             Update the population member of Pelican using Equation (4) 15:             Boundary handing using Equation (10) 16:           ***end*** 17:           Update the best candidate solution 18:           t=t+1 19:         ***end*** 20: ***end for*** 21: ***Output***$\;\mathrm{best}\;\mathrm{candidate}\;\mathrm{solution}\;\left(X_{best}\right)$



## 3. Experimental Results and Analysis

### 3.1. Test Function and Compare Algorithms Parameter Settings

This section evaluates the performance of the proposed ACPOA using the challenging numerical optimization benchmark test suites CEC2017 [36] and CEC2022 [37], and compares it with other algorithms. The comparison algorithms include Velocity Pausing Particle Swarm Optimization (VPPSO) [38], Improved multi-strategy adaptive Grey Wolf Optimizer (IAGWO) [39], Multi-population Evolution Whale Optimization Algorithm (MEWOA) [40], Crayfish Optimization Algorithm (COA) [41], Dung Beetle Optimizer (DBO) [16], Gold Rush Optimizer [42], Crested Porcupine Optimizer (CPO) [18], Artificial rabbits optimization (ARO) [43], and Pelican Optimization Algorithm (POA) [28]. The parameter settings for the algorithms are detailed in Table 1. To ensure fairness and eliminate randomness effects, all algorithms were configured with the same parameters: a population size of 30 and a maximum of 500 iterations. Each algorithm was independently executed 30 times. The experimental results report the mean, standard deviation (Std), and ranking (Rank), with the best results highlighted in bold.

All experiments were conducted under the following computational environment: Windows 10 operating system, equipped with a 13th generation Intel (R) Core (TM) i5-13400 processor (2.5 GHz), 16 GB of RAM, and MATLAB 2024b as the software platform. This unified experimental setup and statistical methodology ensure the reliability and comparability of the results.

### 3.2. Ablation Experiment

**Figure 4:**
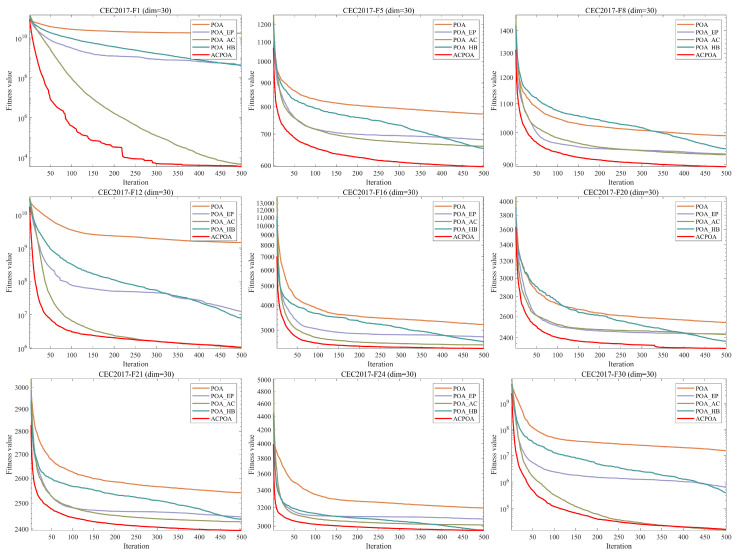
Convergence curves of POA improved with different strategies.

**Figure 5:**
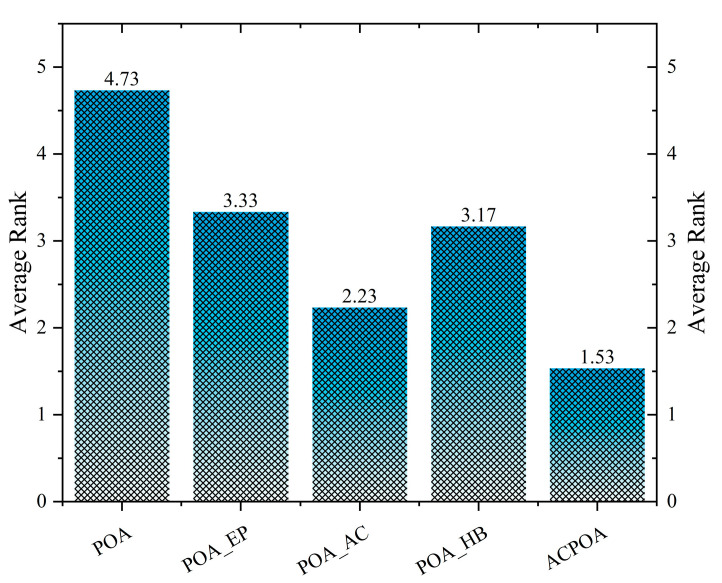
The average ranking of POA improved with different strategies.

To evaluate the independent contributions and synergistic effects of the three enhancement strategies—Elite-pool mutation strategy, Adaptive cooperative mechanism, and Hybrid boundary handling—an ablation study is conducted using the CEC2017 benchmark test set with dimension D=30. Four comparative variants are designed: POA_EP (integrating only the Elite-pool mutation strategy), POA_AC (integrating only the Adaptive cooperative mechanism), POA_HB (integrating only the Hybrid boundary handling), and ACPOA (incorporating all three strategies). The impact of each strategy is assessed through convergence curves (Figure 4) and average rankings (Figure 5).

From Figure 4, it can be observed that the original POA converges slowly on most functions and is prone to local optima; for instance, in F12, the fitness value stagnates after 200 iterations. In contrast, POA_EP consistently outperforms the baseline, reaching stability on F1 after 100 iterations and reducing the final fitness of F12 by approximately 30%, demonstrating the guiding effect of the elite-pool mutation strategy on the search direction. POA_AC shows a clear advantage in high-dimensional functions such as F20, where after 150 iterations its fitness is lower than that of POA_EP, effectively addressing the dimensional coupling issue in the original POA. POA_HB performs better on boundary-sensitive functions such as F8, reducing the final fitness by about 25% after 250 iterations compared with the original POA, thereby effectively preserving boundary search information. ACPOA achieves the best performance across all functions: in F1, it approaches the global optimum within 80 iterations; in F12, no stagnation is observed; and in F24, the convergence is the smoothest. These results confirm that the three strategies synergistically form a complete search framework of “global exploration—local exploitation—boundary optimization.”

Figure 5 presents the average rankings of all algorithmic variants based on the Friedman test, where a smaller rank indicates better overall performance. The original POA records the worst average rank of 4.73. POA_HB achieves an average rank of 3.33, about a 30% improvement over the original POA, though its benefits are limited to boundary optimization. POA_AC ranks slightly better at 3.17, showing stronger adaptability in high-dimensional problems. POA_EP achieves an average rank of 2.23, a 53% improvement over the original POA, and stands out as the most effective single strategy. Finally, ACPOA attains the best rank of 1.53, a 68% improvement over the original POA, significantly outperforming all single-strategy variants. This demonstrates that the three strategies do not merely add up but instead form a closed-loop optimization mechanism of “elite guidance—dimensional cooperation—boundary preservation”, thereby jointly enhancing algorithmic performance.

### 3.3. Assessing Performance with CEC2017 and CEC2022 Test Suite

**Figure 6:**
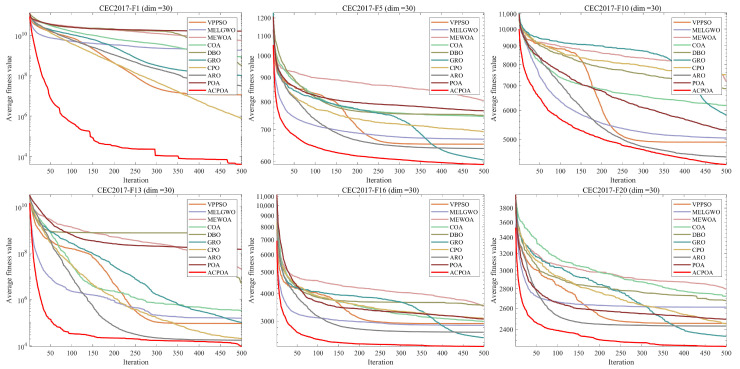

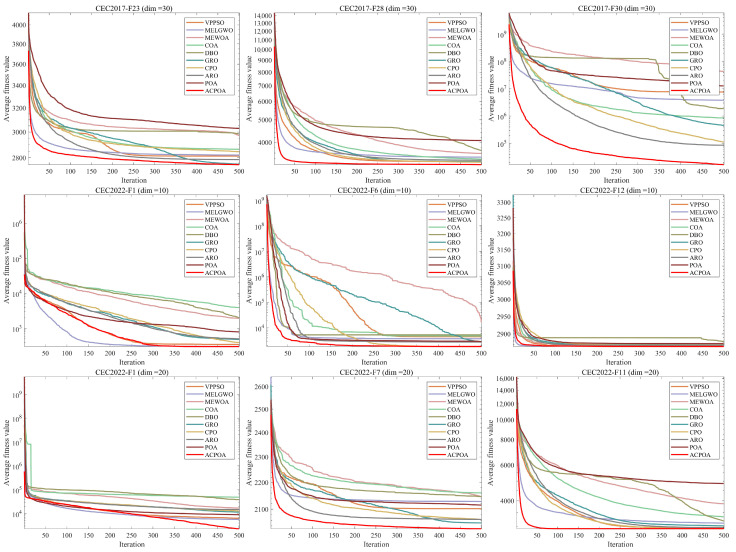
Convergence curves obtained by different algorithms.

In this subsection, the performance of ACPOA is evaluated using the CEC2017 (dimension = 30) and CEC2022 (dimensions = 10/20) benchmark suites. The experimental statistical results are presented in Table 2, Table 3 and Table 4, which detail the mean (Mean) and standard deviation (Std) achieved by each algorithm. The best-performing results are highlighted in boldface. Additionally, convergence curves are illustrated in Figure 6.

The experimental results on the CEC2017 (dim = 30), CEC2022 (dim = 10), and CEC2022 (dim = 20) benchmark suites (Table 2, Table 3 and Table 4 and Figure 6) fully demonstrate the superior performance of ACPOA.

For the CEC2017 (30-dimensional) benchmark suite, the data in Table 2 shows that ACPOA achieves the best mean and standard deviation values in most functions. For example, on function F1, ACPOA attains a mean value of 3.9708 × 10^3^, which is significantly lower than POA’s 1.6176 × 10^10^ and VPPSO’s 1.1375 × 10^7^, with a smaller standard deviation, indicating both strong optimization capability and stability in unimodal high-dimensional problems. On function F9, ACPOA achieves a mean of 1.4125 × 10^3^, outperforming DBO’s 6.9243 × 10^3^ and others, demonstrating its advantage in handling multimodal problems. According to the Friedman ranking, ACPOA ranks first with an average rank of 1.47, leading other algorithms. The convergence curves for this benchmark suite (e.g., F1, F5, F10) shown in Figure 1 indicate that ACPOA’s fitness rapidly decreases in the early iterations and stabilizes sooner, whereas MEWOA, MELGWO, and others converge more slowly. Specifically, for function F1, ACPOA approaches the optimum within 100 iterations, while other algorithms remain in higher-value regions, confirming its fast convergence capability.

For the CEC2022 (10-dimensional) benchmark suite, Table 3 reveals a clear advantage of ACPOA. On function F3, ACPOA achieves the theoretical optimum of 6.0000 × 10^2^, comparable to MELGWO and CPO, but with the smallest standard deviation of 1.2467 × 10^−3^, indicating higher convergence precision. On function F6, ACPOA’s mean value is 1.8339 × 10^3^, close to CPO, but with a standard deviation only 24.5% that of CPO, demonstrating better stability. With an average rank of 2.08, ACPOA ranks first overall. The convergence curve for CEC2022-F1 (dim = 10) in Figure 6 shows that ACPOA’s convergence speed significantly exceeds that of POA and MEWOA, with a more stable trajectory.

For the CEC2022 (20-dimensional) benchmark suite, Table 4 demonstrates the strong scalability of ACPOA in medium-to-high-dimensional scenarios. On function F1, ACPOA achieves a mean value of 2.2912 × 10^3^, which is only 23.1% of POA and 6.1% of WOA, with a lower standard deviation. On function F10, the mean value reaches 2.4814 × 10^3^, significantly lower than those of PSO and GWO, highlighting the effectiveness of elite pool-based information sharing. With an average rank of 1.42, ACPOA again ranks first. The convergence curve for CEC2022-F1 (dim = 20) in Figure 6 further confirms ACPOA’s rapid and stable convergence, which can be attributed to the subpopulation–dimension dynamic allocation mechanism that improves high-dimensional search efficiency.

On CEC2022 (10D vs. 20D), ACPOA’s average rank increases by only 0.66 (from 2.08 to 1.42), while POA’s rank drops by 0.7 (from 7.42 to 8.17) (Table 3 and Table 4). This indicates ACPOA’s adaptive cooperative mechanism reduces dimensional coupling—subgroup-dimension allocation (S = min(4, D)) ensures each subgroup focuses on ≤D/4 dimensions, avoiding “dimension disaster” (Figure 6, CEC2022-F1 (20D) convergence curve: ACPOA’s fitness is 2.2912 × 10^3^, 76.9% lower than POA’s 8.9836 × 10^3^).

For unimodal functions (e.g., CEC2017-F1), ACPOA’s mean fitness is 3.9708 × 10^3^ (POA: 1.6176 × 10^10^) due to elite pool mutation accelerating convergence; for multimodal functions (e.g., CEC2017-F9), ACPOA’s mean (1.4125 × 10^3^) outperforms DBO (6.9243 × 10^3^) because adaptive cooperation maintains diversity; for composite functions (e.g., CEC2017-F30), ACPOA’s low std (1.3243 × 10^4^) reflects hybrid boundary handling preserving boundary information (Table 2).

Moreover, Figure 1 illustrates ACPOA’s “global exploration followed by local exploitation” convergence pattern. For example, in CEC2017-F11, the algorithm initially conducts broad global exploration, then gradually focuses on promising regions for refinement, ultimately converging to superior solutions. This behavior results from the balance between exploration and exploitation achieved through the elite pool-based mutation and adaptive cooperative mechanisms. Overall, ACPOA demonstrates outstanding convergence speed, accuracy, and stability across benchmark suites with different dimensionalities.

**Figure 7:**
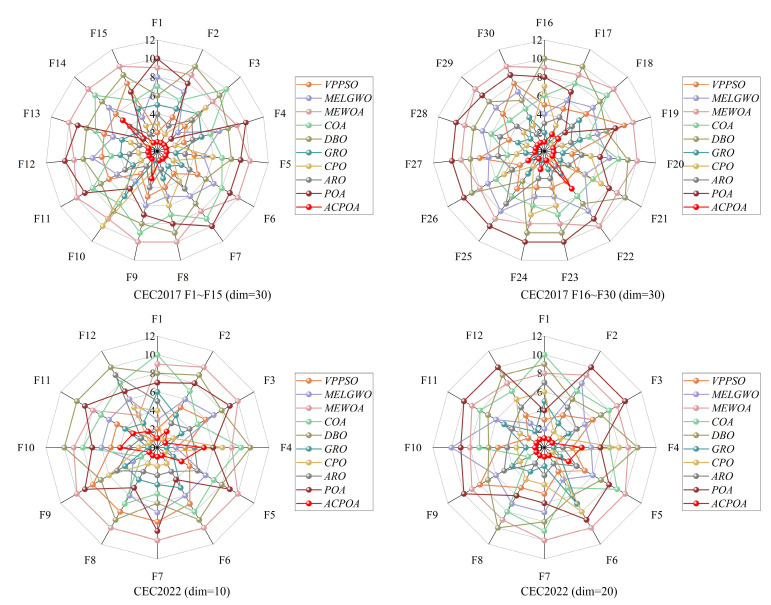
Ranking distribution of different algorithms.

The rank distribution in Figure 7 further verifies the robustness of ACPOA. In the CEC2017 (dim = 30) suite, ACPOA ranks first on 16 out of 30 functions, with particularly strong performance on high-dimensional multimodal functions such as F12 and F15. In the CEC2022 (dim = 10/20) suites, its rankings are consistently concentrated in the top 1–2 positions, whereas algorithms like POA and COA exhibit large rank fluctuations. This indicates that ACPOA is more adaptable to different types of functions and less affected by specific problem characteristics.

**Figure 8:**
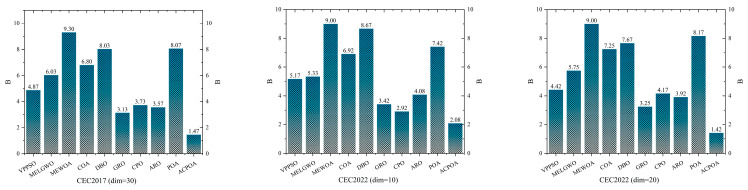
Average rank ranking.

As shown in Figure 8, ACPOA achieves the lowest average ranks across all three benchmark scenarios (1.47, 2.08, and 1.42), with a significant lead over the second-best algorithms. This consistent advantage is attributed to the integration of elite pool mutation, adaptive cooperation, and hybrid boundary handling strategies, which effectively enhance the algorithm’s generality and robustness across varying dimensionalities and problem complexities.

In summary, ACPOA demonstrates superior convergence speed, optimization accuracy, and stability across various benchmark functions, which can be attributed to the integration of three key improvement strategies. First, the elite pool mutation strategy constructs an elite pool composed of the top three individuals with the best fitness values. This enhances global exploration and improves local exploitation accuracy, while preventing premature loss of population diversity—thus reducing the risk of getting trapped in local optima when optimizing complex multimodal functions. Second, the adaptive cooperative learning mechanism employs subpopulation–dimension dynamic allocation and specialized updating to enable task specialization and global information sharing. This significantly improves the search efficiency in high-dimensional spaces and allows the algorithm to better adapt to the varying characteristics of different dimensionalities. Third, the hybrid boundary handling technique maintains population stability while preserving valuable search information. Compared to simple truncation methods, it enables more effective exploration near boundary-optimal regions.

The synergy of these three strategies collectively underpins ACPOA’s performance advantage, which is comprehensively validated through statistical results, convergence curves, and ranking distributions.

### 3.4. Convergence Behavior Analysis

**Figure 9:**
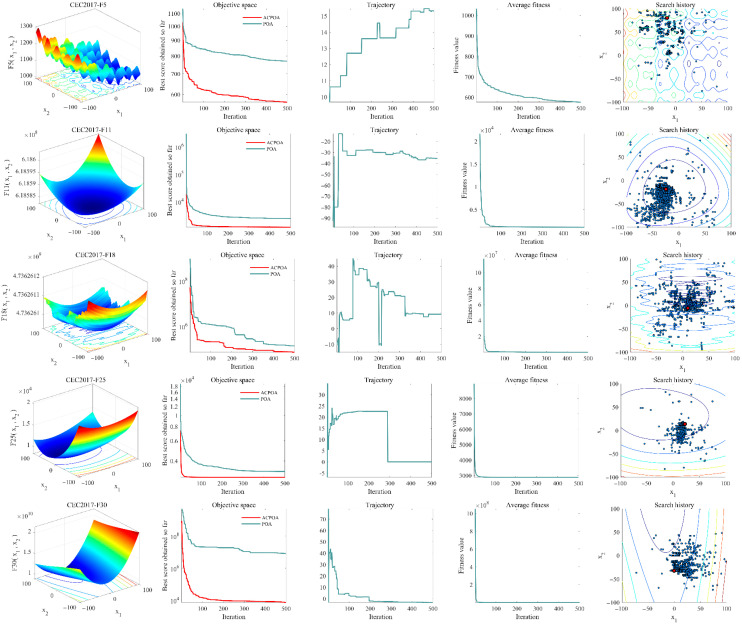
Convergence behavior of ACPOA and POA.

Figure 9 systematically illustrates the performance enhancement of ACPOA over the standard POA through the average fitness history curves, search trajectories, convergence curves, and position variation visualizations.

In the average fitness history curves (second column), ACPOA consistently maintains lower fitness values than POA throughout the iterations, with the performance gap gradually widening. For instance, in CEC2017-F25, ACPOA’s fitness value drops to less than 50% of that of POA within the first 100 iterations and continues to approach the global optimum in subsequent steps. This improvement is largely attributed to the elite pool mutation strategy, which—with a 10% probability of using the elite mean and 90% probability of randomly selecting elite individuals—enhances the directionality of the population toward the global optimum and avoids the slow convergence issue caused by random perturbations in POA.

The search trajectories (third column) reveal ACPOA’s characteristic of “broad early exploration followed by fine-tuned exploitation.” As shown in CEC2017-F11, the algorithm initially explores a wide range along the first dimension (exploration phase) and later focuses on minor adjustments within promising regions (exploitation phase). This dynamic balance results from the adaptive cooperative learning mechanism, where dimension-specific updates by subgroups preserve diversity during global exploration and improve precision in local exploitation. In contrast, POA’s unified update strategy often leads to premature stagnation in local areas.

The convergence curves (fourth column) further confirm ACPOA’s fast convergence ability. For functions such as CEC2017-F1 and F10, ACPOA exhibits steeper curves, reaching stable values with fewer iterations. For example, in F1, it converges near the global optimum within 200 iterations, whereas POA requires more than 300 iterations and still yields a higher final fitness value. This advantage stems from the hybrid boundary handling technique, which incorporates a 40% tendency toward elites, 40% mirror reflection, and 20% random reinitialization. This design reduces interference from boundary-violating individuals, preserves useful information, and accelerates convergence.

The position variation visualizations (fifth column) indicate that ACPOA’s search paths are more targeted. Initially, solutions are uniformly distributed across the search space (ensuring exploration breadth), then gradually converge toward the region near the global optimum (improving exploitation accuracy). For instance, in F11, the position distribution transitions from scattered to concentrated, whereas POA exhibits irregular aggregation patterns that often lead to premature convergence. These results validate the synergistic effects of the three core strategies: the elite pool provides high-quality guidance, the adaptive cooperative mechanism optimizes search roles, and the hybrid boundary technique maintains population stability—all working together to enable ACPOA’s superior performance on complex optimization problems.

### 3.5. Computing Time Analysis

**Figure 10:**
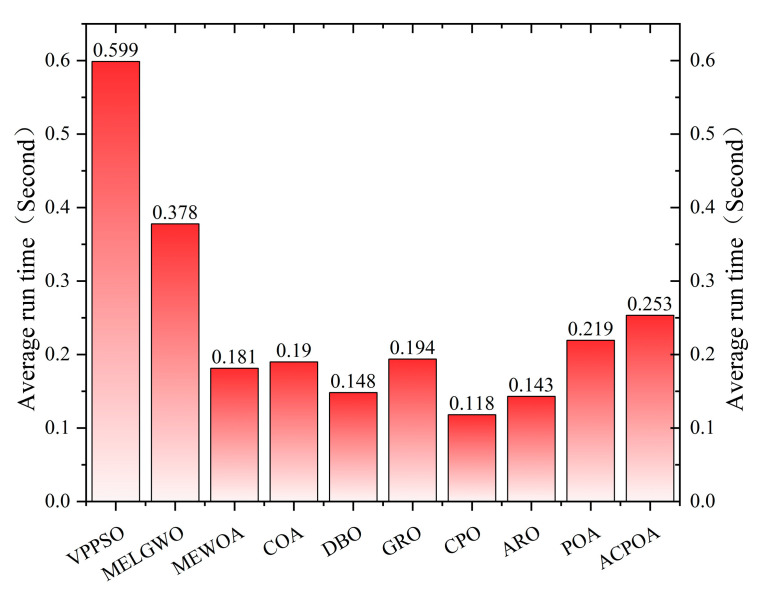
Average computation time of different algorithms.

Computation time is one of the key indicators for evaluating the practicality of optimization algorithms. In scenarios such as image segmentation and engineering optimization, where real-time performance is crucial, algorithmic efficiency directly determines its application value. To quantitatively assess the computational cost of ACPOA, it is first noted that prior experimental results have confirmed the overall superiority of ACPOA compared with the traditional POA in terms of optimization performance. This section therefore provides a more detailed comparative analysis of their computational costs, with a particular focus on differences in execution time. To ensure fairness, all algorithmic parameters were standardized: the population size was set to 30, the maximum number of iterations was fixed at 500, and each algorithm was independently executed 30 times. Figure 10 illustrates the average computation time (in seconds) of the compared algorithms when solving the CEC2017 test functions (D = 30).

The figure presents the average running time of each algorithm (in seconds), with ACPOA recording 0.253 s. In comparison, algorithms such as VPSO (0.599 s) and MELGWO (0.378 s) incur much higher costs due to their complex population interaction mechanisms. CPO (0.219 s) shows a runtime close to that of ACPOA, reflecting a similar trade-off between computational overhead and performance improvement. Algorithms such as COA (0.181 s), DBO (0.190 s), GRO (0.148 s), ARO (0.118 s), and POA (0.194 s) exhibit shorter runtimes owing to their relatively streamlined processes or efficient resource utilization.

Overall, although ACPOA is not the most time-efficient algorithm, it achieves significant improvements in optimization accuracy and convergence through strategies such as elite-pool mutation and adaptive cooperation. In practical scenarios where solution quality is prioritized, the additional computational cost remains acceptable. Therefore, the choice of algorithm should be made by balancing execution efficiency with optimization effectiveness.

### 3.6. Time Complexity Analysis

Time complexity is a key metric to evaluate the efficiency of optimization algorithms, which reflects the growth trend of computational overhead with the increase of problem scale (e.g., population size N, number of dimensions dim, maximum iterations T). This section analyzes the time complexity of ACPOA and 9 comparison algorithms (VPPSO, MELGWO, MEWOA, COA, DBO, GRO, CPO, ARO, POA) based on their core search mechanisms, and the results are shown in Table 5.

All algorithms have the same asymptotic time complexity (O (T × N × dim)) because they are all population-based iterative optimization algorithms. The difference lies in the constant term (hidden in the big O notation), where ACPOA introduces a small constant overhead due to the three improvement strategies, but this overhead is negligible compared to the dominant term (T × N × dim), which is consistent with the average computation time results in Section 3.5.

## 4. Experimental Results for Multilevel Thresholding

In image threshold segmentation, two commonly used methods for determining the optimal threshold are the Otsu method and the Kapur entropy method. For different thresholds, the variance between the target and background regions varies. The optimal threshold ($T_{best}$) is the one that maximizes this variance. The Kapur method calculates the sum of the entropies of the segmented target and background regions based on their probability distributions and selects the threshold that maximizes this sum. Both methods take the image pixels as input and determine the optimal threshold based on their respective computational principles [6,44,45]. In this study, we adopt the Otsu method to find the optimal threshold.

The Otsu method determines the optimal threshold based on the between-class variance criterion. Suppose the image I contains a total of L gray levels, and the number of pixels with gray level i is $n_{i}$, Then, the total number of pixels in the image is:

(11) $$\begin{array}{c} \;\;\;\;\;\;\;\;\;\;\;\;\;\;\;\;\;\;\;\;\;\;\;\;\;\;\;\;\;N=\sum_{i=0}^{L-1}n_{i} \end{array}$$

The probability of a pixel having gray level i is:

(12) $$\begin{array}{c} \;\;\;\;\;\;\;\;\;\;\;\;\;\;\;\;\;\;\;\;\;\;\;\;\;\;\;\;\;\;\;P_{i}=\frac{n_{i}}{N},i=0,1,\ldots ,L-1 \end{array}$$

where $P_{i}\geq 0,and\;P_{0}+P_{1}+\cdots +P_{L-1}=1$.

Let the number of thresholds be k. If a gray value t is chosen as the threshold, the image is divided into two regions: the pixels with gray levels in [0, t] are considered the foreground (target), and those in [t+1, L−1] are the background. If the proportion of target pixels in the entire image is $\omega_{0}$ with an average gray level of $\mu_{0}$,and the proportion of background pixels is $\omega_{1}$ with an average gray level of $\mu_{1}$,then the total mean gray level of the image is μ, and the between-class variance is denoted by v. The formulas for $\omega_{0}$, $\mu_{0}$, $\omega_{1}$, $\mu_{1}$, μ and v are as follows [46]:

(13) $$\omega_{0}=\sum_{i=0}^{t}P_{i}\;\mu_{0}=\sum_{i=0}^{t}\frac{iP_{i}}{\omega_{0}}\;\omega_{1}=\sum_{i=t+1}^{L-1}P_{i}\;\mu_{1}=\sum_{i=t+1}^{L-1}\frac{iP_{i}}{\omega_{1}}\;\mu =\sum_{i=0}^{L-1}iP_{i}\;(t)=\omega_{0}{\left(\mu_{0}-\mu \right)}^{2}+\omega_{1}{\left(\mu_{1}-\mu \right)}^{2}=\omega_{0}\omega_{1}{\left(\mu_{0}-\mu_{1}\right)}^{2}$$

The formula for calculating the optimal threshold $T_{best}$ is:

(14) $$\begin{array}{c} \;\;\;\;\;\;\;\;\;\;\;\;\;\;\;\;\;\;\;\;\;\;\;\;\;\;\;\;\;\;\;t_{\mathrm{best}\;}=\underset{0\leq t\leq L-1}{\arg max}\left[v(t)\right] \end{array}$$

By extension, the between-class variance for k thresholds can be calculated using the following formula:

(15)                     v(t1,t2,…,tk)=ω0ω1(μ0−μ1)2+ω0ω2(μ0−μ2)2+⋯+ω0ωk(μ0−μk)2+ω1ω2(μ1−μ2)2+⋯+ω1ω3(μ1−μ3)2+⋯+ωk−1ωk(μk−1−μk)2

where the formulas for $\omega_{i}$ and $\mu_{i}$ are given as:

(16)                         ωi−1=∑i=ti−1+1tiPi,1≤i≤k+1μi−1=∑i=ti−1+1tiiPiωi−1,1≤i≤k+1

Let the optimal threshold set based on Equation (14) be denoted as $T_{best}$, then it can be calculated by:

(17) $$\begin{array}{c} \;\;\;\;\;\;\;\;\;\;\;\;\;\;\;\;\;\;\;\;\;\;\;\;\;\;\;\;\;\;\;\;T_{best}=\underset{0\leq t_{1}\leq t_{2}\leq \ldots \leq t_{k}}{\arg max}[v(t_{1},t_{2},\cdots ,t_{k})] \end{array}$$

**Figure 11:**
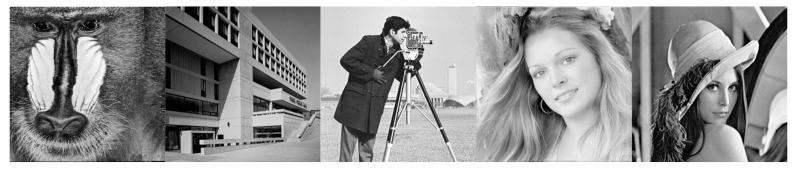
The set of benchmark images.

In this study, comparative experiments were conducted to validate the performance of the ACPOA algorithm in multi-level thresholding. Five improved population intelligence algorithms were selected as baselines. All algorithms were configured with a unified population size of N = 30 and a maximum number of iterations T = 25. The Otsu method was employed as the objective function. Experiments were carried out on five benchmark images with varying styles, using four different numbers of thresholds (2, 4, 6, and 8), as illustrated in Figure 11. Each set of experiments was independently run 30 times. The mean (Mean) and standard deviation (Std) of the objective function values were recorded to evaluate the accuracy and stability of each algorithm. To ensure fairness in comparison, all algorithm parameters were kept consistent with those described earlier.

### 4.1. Evaluation Index

The evaluation metrics used to assess image segmentation performance include Peak Signal-to-Noise Ratio (PSNR), Structural Similarity Index Measure (SSIM), and Feature Similarity Index Measure (FSIM). Higher values of PSNR and SSIM indicate lower distortion and better visual quality of the segmented image. Similarly, a higher FSIM value corresponds to lower error rates. The formulas for these evaluation metrics are as follows [6,47]:

The PSNR is calculated as:

(18) $$\begin{array}{c} \;\;\;\;\;\;\;\;\;\;\;\;\;\;\;\;\;\;\;\;\;\;\;\;\;\;\;\;\;\;\;\;\;\mathrm{PSNR}=10{log}_{10}(\frac{255^{2}}{MSE}) \end{array}$$

where MSE is the Mean Squared Error between the original image I and the segmented image $I^{\prime }$. The MSE is defined as:

(19) $$\begin{array}{c} \;\;\;\;\;\mathrm{MSE}=\frac{1}{MN}\sum_{j=1}^{M}\sum_{k=1}^{N}{\left[I(j,k)-I^{\prime }(j,k)\right]}^{2} \end{array}$$

where (M×N) is the image size, I and $I^{\prime }$ are the original and segmented images, respectively, and I(j,k) represent the pixel intensities at position (j,k) in the original and segmented grayscale images.

SSIM evaluates the similarity between two images by comparing their luminance, contrast, and structural information. Given two images I and $I^{\prime }$, SSIM is defined as:

(20) $$\begin{array}{c} \;\;\;\;\;\;\;\;\;\;\;\;\;\;\;\;\;\;\;\;\;\mathrm{SSIM}(I,I^{\prime })=\frac{\left(2\mu_{I}\mu_{I^{\prime }}+C_{1})(2\sigma_{{II}^{\prime }}+C_{2}\right)}{\left(\mu_{I}^{2}+\mu_{I^{\prime }}^{2}+C_{1})(\sigma_{I}^{2}+\sigma_{I^{\prime }}^{2}+C_{2}\right)} \end{array}$$

where $\mu_{I}$ and $\mu_{I^{\prime }}$ are the mean pixel intensities of I and $I^{\prime }$, respectively; $\sigma_{{II}^{\prime }}$ is the covariance between I and $I^{\prime }$; $\sigma_{I}^{2}$ and $\sigma_{I^{\prime }}^{2}$ are the variances of I and $I^{\prime }$, respectively. Constants $C_{1}$ and $C_{2}$ are used to stabilize the division and are typically defined as $C_{1}=K_{1}L$, $C_{2}={\left(K_{2}L\right)}^{2}$, where $K_{1}=0.01$, $K_{2}=0.03$, and L is the maximum grayscale intensity value.

The Structural Similarity Index (SSIM) ranges from −1 to 1, reaching its maximum value of 1 when two images are identical. This index is grounded in human visual perception and decomposes image quality into three independent components: luminance, contrast, and structural information. Luminance is estimated using local means, contrast is characterized by standard deviation, and structure similarity is measured via covariance. By evaluating these three factors separately, SSIM effectively quantifies the level of distortion in an image. A higher SSIM value, closer to 1, indicates better structural preservation, which reflects superior segmentation performance in image processing tasks [11,48].

Since perceptually significant image features are typically located at points of high phase consistency, FSIM adopts phase congruency as the primary feature for evaluating image quality. As phase congruency is not affected by contrast—a factor that often influences image quality—FSIM introduces image gradient as a secondary feature to enhance the quality assessment [6].

Let $e_{n}$ and $o_{n}$ represent the even-symmetric and odd-symmetric filters at scale n, respectively, which form an orthogonal pair. At a point x,each orthogonal pair forms a corresponding vector $\left[e_{n}(x),o_{n}(x)\right]$, and the amplitude at scale n is given by $A_{n}(x)=\sqrt{e_{n}(x{)}^{2}+o_{n}(x{)}^{2}}$. Assuming $E(x)=\sqrt{{\left(\sum_{n}e_{n}(x)\right)}^{2}+{\left(\sum_{n}o_{n}(x)\right)}^{2}}$, the phase congruency (PC) for a one-dimensional signal is defined as:

(21) $$\begin{array}{c} \;\;\;\;\;\;\;\;\;\;\;\;\;\;\;\;\;\;\;\;\;\;\;\;\;\;\;PC(x)=\frac{E(x)}{\varepsilon +\sum_{n}A_{n}(x)} \end{array}$$

where ε is a small positive constant to avoid division by zero.

For quadrature filters, the LogGabor filter is commonly used. In the frequency domain, the transfer function of a LogGabor filter is defined as:

(22) $$\begin{array}{c} \;\;\;\;\;\;\;\;\;\;\;\;\;\;\;\;\;\;\;\;\;\;\;\;\;\;\;\;\;\;\;\;\;\;\;G(\omega )=exp\left(-\frac{{\left(log(\omega /\omega_{0})\right)}^{2}}{2\sigma_{r}^{2}}\right) \end{array}$$

where $\omega_{0}$ is the center frequency of the filter, and $\sigma_{r}$ controls the bandwidth.

To extend the one-dimensional LogGabor filter into two dimensions, a spreading function is applied in the vertical direction. When a Gaussian function is used as the spreading function, the transfer function of the 2D LogGabor filter is given by:

(23) $$\begin{array}{c} \;\;\;\;\;\;\;\;\;\;\;G_{2}(W,\theta_{j})=exp\left(-\frac{{\left(log(\omega /\omega_{0})\right)}^{2}}{2\sigma_{r}^{2}}\right)\times exp\left(-\frac{{\left(\theta -\theta_{j}\right)}^{2}}{2\sigma_{\theta }^{2}}\right) \end{array}$$

where, $\theta_{j}=j\pi /J,j=\{0,1,\cdots ,J-1\}$ denotes the orientation angles of the filters, J is the total number of orientations, and $\sigma_{\theta }$ is the angular bandwidth.

By adjusting $\omega_{0}$ and $\theta_{j}$, and convolving the 2D filter $G_{2}$ with the input image, each pixel yields a response denoted as $\left[e_{n,\theta_{j}}(x),o_{n,\theta_{j}}(x)\right]$. The amplitude in direction $\theta_{j}$ is computed as:

(24) $$\begin{array}{c} \;\;\;\;\;\;\;\;\;\;\;\;\;\;\;\;\;\;\;\;\;\;\;\;\;A_{n,\theta_{j}}(x)=\sqrt{e_{n,\theta_{j}}(x{)}^{2}+o_{n,\theta_{j}}(x{)}^{2}} \end{array}$$

The directional energy in direction $\theta_{j}$ is defined as:

(25) $$\begin{array}{c} \;\;\;\;\;\;\;\;\;\;\;\;\;\;\;\;\;\;\;\;\;\;\;\;E_{\theta_{j}}(x)=\sqrt{F_{\theta_{j}}(x{)}^{2}+H_{\theta_{j}}(x{)}^{2}} \end{array}$$

where $F_{\theta_{j}}(x)=\sum_{n}e_{n,\theta_{j}}(x),H_{\theta_{j}}(x)=\sum_{n}o_{n,\theta_{j}}(x)$. Consequently, the 2D phase congruency at pixel xxx is computed as:

(26) $$\begin{array}{c} \;\;\;\;\;\;\;\;\;\;\;\;\;\;\;\;\;\;\;\;\;\;\;\;\;PC_{2D}\left(x\right)=\frac{\sum_{j}E_{\theta_{j}}(x)}{\varepsilon +\sum_{n}\sum_{j}A_{n,\theta_{j}}(x)} \end{array}$$

The gradient can be represented using convolution kernels, with the three most commonly used methods for computing gradients being the Sobel operator, Prewitt operator, and Scharr operator. The gradient magnitude (GM) of an image is defined as $G={\left(G_{x}^{2}+G_{y}^{2}\right)}^{0.5}$, where $G_{x}$ and $G_{y}$ represent the gradients computed in the horizontal and vertical directions, respectively.

After obtaining phase consistencies ${PC}_{1}$ and ${PC}_{2}$, where ${PC}_{1}$ and ${PC}_{2}$ are the phase consistency values extracted from two images, and $G_{1}$ and $G_{2}$ are their corresponding gradient magnitudes, the image quality metrics $S_{PC}\left(x\right)$ and $S_{GM}\left(x\right)$ are calculated as follows:

(27) $$\begin{array}{c} \;\;\;\;\;\;\;\;\;\;\;\;\;\;\;\;\;\;\;\;\;\;\;\;\;S_{PC}(x)=\frac{2PC_{1}(x)\cdot PC_{2}(x)+T_{1}}{PC_{1}^{2}(x)+PC_{2}^{2}(x)+T_{1}} \end{array}$$

(28) $$\begin{array}{c} \;\;\;\;\;\;\;\;\;\;\;\;\;\;\;\;\;\;\;\;\;\;\;\;\;\;\;\;\;S_{GM}(x)=\frac{2G_{1}(x)\cdot G_{2}(x)+T_{2}}{G_{1}^{2}(x)+G_{2}^{2}(x)+T_{2}} \end{array}$$

where, $T_{1}$ and $T_{2}$ are small positive constants introduced for numerical stability, and $G_{1}$ and $G_{2}$ correspond to the gradient magnitudes of the original and segmented images, respectively.

By combining $S_{PC}\left(x\right)$ and $S_{GM}\left(x\right)$,the similarity measure $S_{L}\left(x\right)$ between the reference image and the distorted image can be obtained:

(29) $$\begin{array}{c} \;\;\;\;\;\;\;\;\;\;\;\;\;\;\;\;\;\;\;\;\;\;\;\;\;\;\;\;\;\;\;\;\;S_{L}(x)=={\left[S_{PC}(x)\right]}^{\alpha }{\left[S_{G}(x)\right]}^{\beta } \end{array}$$

where α and β are parameters for adjustment, typically set to 1.

The Feature Similarity Index (FSIM) between the reference image I and the distorted image $I^{\prime }$ is calculated as:

(30) $$\begin{array}{c} \;\;\;\;\;\;\;\;\;\;\;\;\;\;\;\;\;\;\;\;\;\;\;\;\;\;\;\;\;\;\;FSIM(I,I^{\prime })=\frac{\sum_{x\in \Omega }S_{L}(x)\cdot PC_{m}(x)}{\sum_{x\in \Omega }PC_{m}(x)} \end{array}$$

in this equation, x represents a pixel location, Ω denotes the entire image domain, and $PC_{m}(x)=max(PC_{1}(x),PC_{2}(x))$, where $PC_{1}(x)$ and $PC_{2}(x)$ are the phase consistency values of the original and segmented images, respectively.

### 4.2. Analysis of Otsu Results Based on ACPOA

Using the ACPOA algorithm with Otsu’s method as the objective function, multilevel thresholding segmentation was performed on five selected images. The effectiveness of the thresholding algorithm is reflected by the maximum value of the Otsu objective function, as well as the PSNR, FSIM, and SSIM metrics. Higher values of these three evaluation indicators indicate better image segmentation performance.

The results demonstrate that the proposed ACPOA algorithm outperforms other algorithms in terms of the objective function value, PSNR, FSIM, and SSIM. Table 6 presents the distribution of the optimally selected thresholds on the histograms. Table 7 shows the mean and standard deviation of the best fitness values obtained by each algorithm using Otsu’s objective function, along with the average ranking results for each algorithm. Table 8, Table 9 and Table 10 report the mean and standard deviation of PSNR, FSIM, and SSIM values obtained by each algorithm, respectively, as well as their average ranks. Optimal thresholds were searched using Otsu’s objective function with threshold numbers set to 2, 4, 6, and 8.

**Figure 12:**
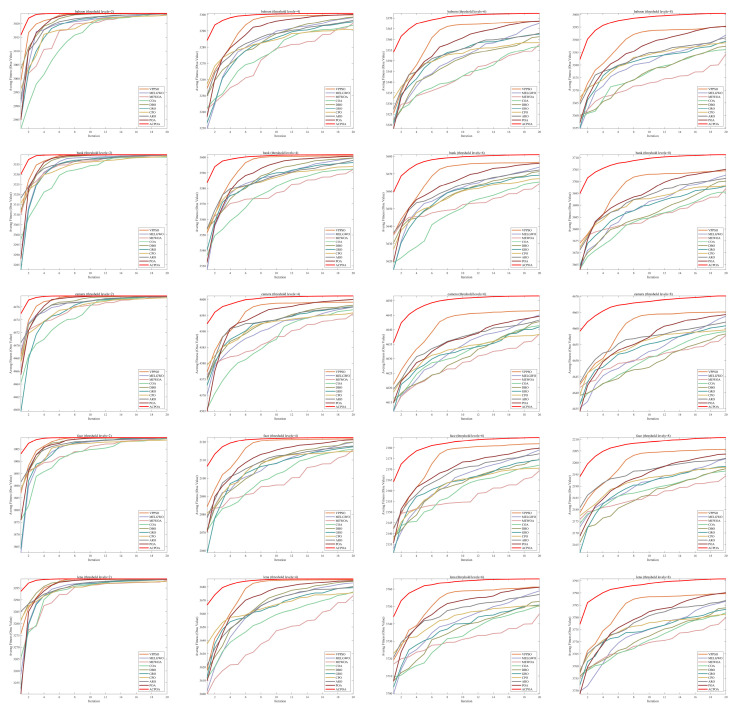
Otsu fitness value curves of different algorithms.

Figure 12 displays the Otsu fitness value curves of different algorithms. These curves allow for a comparison of the convergence speed and the final fitness values achieved by various algorithms. As observed in the figure, the standard POA and other algorithms show a relatively small gap compared to ACPOA when the threshold is set to 2. However, as the threshold increases, the advantage of ACPOA becomes increasingly evident, with its fitness value rising most significantly and eventually stabilizing at a higher level. Compared to the curves of other algorithms, the ACPOA algorithm not only converges faster, rapidly improving the fitness value with fewer threshold increments, but also achieves the highest final fitness value. This indicates that as the threshold increases, the proposed ACPOA algorithm performs better in optimizing the Otsu fitness function, demonstrating the best fitness value performance.

**Figure 13:**
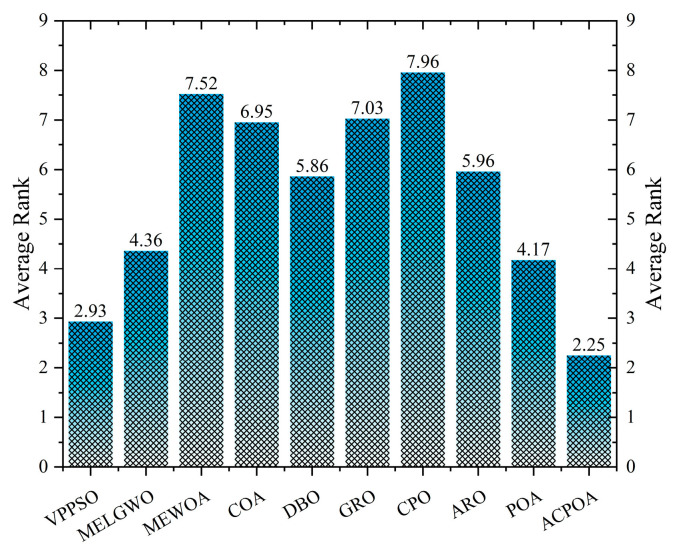
Average rank ranking of the fitness values in Otsu.

Table 7 presents the mean and standard deviation of the best fitness values obtained by different algorithms for multilevel thresholding (2, 4, 6, and 8 levels) using Otsu’s objective function on five images (baboon, bank, camera, face, and lena). The results show that ACPOA demonstrates superior performance in almost all cases. For instance, in the 8-level thresholding of the baboon image, ACPOA achieved a fitness mean of 3.40 × 10^3^, slightly higher than POA’s 3.39 × 10^3^. More importantly, its standard deviation was only 1.70 × 10^−1^, significantly lower than POA’s 2.12 × 10^0^, indicating that ACPOA not only finds better threshold combinations but also exhibits greater stability across repeated experiments. Similarly, for the 6-level thresholding of the camera image, ACPOA’s mean fitness value was 4.65 × 10^3^, equal to that of POA. However, the standard deviation for ACPOA (7.40 × 10^−2^) was markedly lower than POA’s 2.72, demonstrating that the elite pool mutation strategy effectively guides the population toward high-quality regions, thereby reducing performance fluctuations caused by random disturbances. As shown in Figure 13, ACPOA ranks first with an average rank of 2.25, significantly outperforming POA, which ranks second at 4.17. This advantage is especially pronounced at higher threshold levels (6 and 8), indicating that ACPOA maintains efficient optimization capability even in complex threshold spaces.

**Figure 14:**
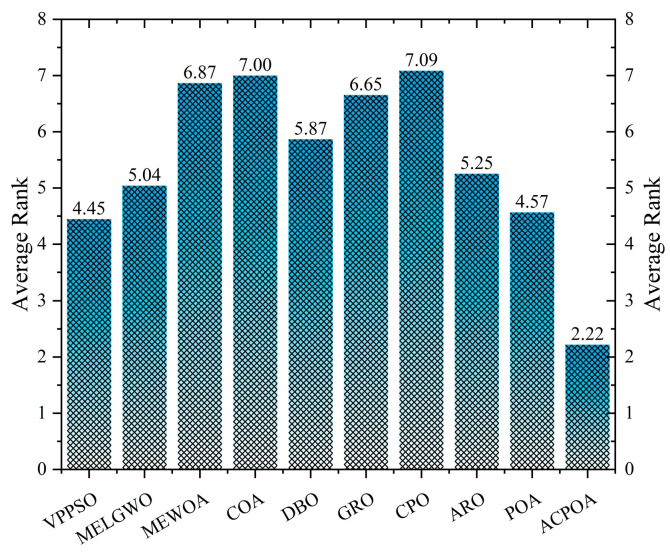
Average rank of PSNR in Otsu.

Table 8 records the Peak Signal-to-Noise Ratio (PSNR) results of image segmentation from different algorithms. PSNR reflects the distortion level between the segmented and original images, with higher values indicating better performance. ACPOA demonstrates significant advantages across all scenarios. For example, in the 8-level thresholding of the bank image, ACPOA achieves a mean PSNR of 25.2639, outperforming POA (24.7618), MELGWO (24.6335), and VPPSO (24.7295). Moreover, its standard deviation (5.18 × 10^−2^) is the lowest, indicating segmentation results closer to the original image with better detail preservation. In the 6-level thresholding of the face image, ACPOA attains a PSNR of 22.5815, superior to POA’s 22.4368 and MEWOA’s 21.8273. As shown in Figure 14, ACPOA ranks first with an average rank of 2.22, significantly ahead of MEWOA (6.87) and standard POA (4.57). This advantage is especially notable at higher threshold levels (6 and 8), highlighting ACPOA’s ability to maintain efficient optimization in complex threshold spaces. This success is attributed to the adaptive cooperative mechanism, which, through specialized subgroup division of labor, precisely locates the optimal solutions in high-dimensional threshold spaces and mitigates the search inefficiency caused by dimensional coupling. Additionally, ACPOA’s PSNR values in 8-level thresholding are generally higher than those in lower-level scenarios, with its superiority increasing as the number of thresholds grows, confirming its adaptability in complex segmentation tasks.

**Figure 15:**
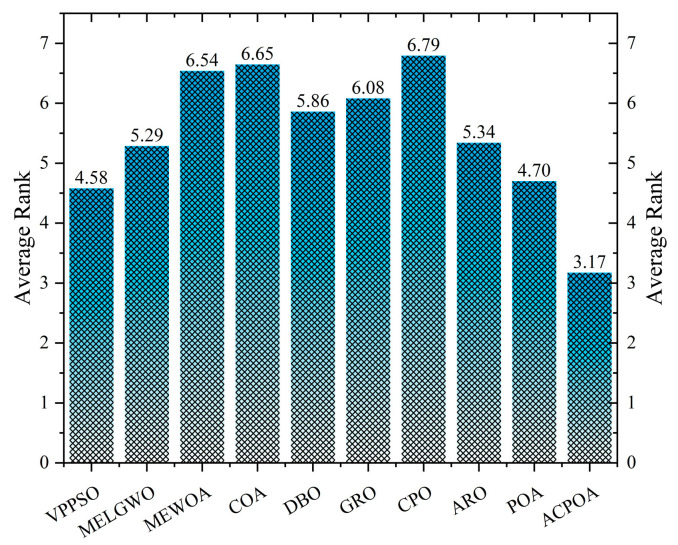
Average rank of FSIM in Otsu.

Table 9 presents the Feature Similarity Index (FSIM) results, which evaluate how well the segmented image retains the original image’s features such as edges and textures. Higher FSIM values indicate better feature consistency. ACPOA stands out in this regard. For instance, in the 8-level thresholding of the lena image, ACPOA achieves a mean FSIM of 0.8840, considerably higher than POA (0.8741), VPPSO (0.8706), and MELGWO (0.8685). This reflects the effectiveness of the hybrid boundary handling strategy in preserving more meaningful feature information during threshold search. In the 6-level thresholding of the baboon image, ACPOA’s FSIM reaches 0.8861, slightly surpassing POA’s 0.8859, with a standard deviation (2.54 × 10^−3^) only 23% of POA’s, demonstrating greater stability in feature retention across repeated experiments. Similarly, as shown in Figure 15, ACPOA ranks first with an average rank of 3.17. Notably, ACPOA’s advantage is more pronounced in texture-rich images such as baboon and lena. This is attributed to the adaptive cooperative mechanism dynamically allocating dimensions so that subgroups focus on capturing thresholds corresponding to different features, thereby enhancing feature similarity.

**Figure 16:**
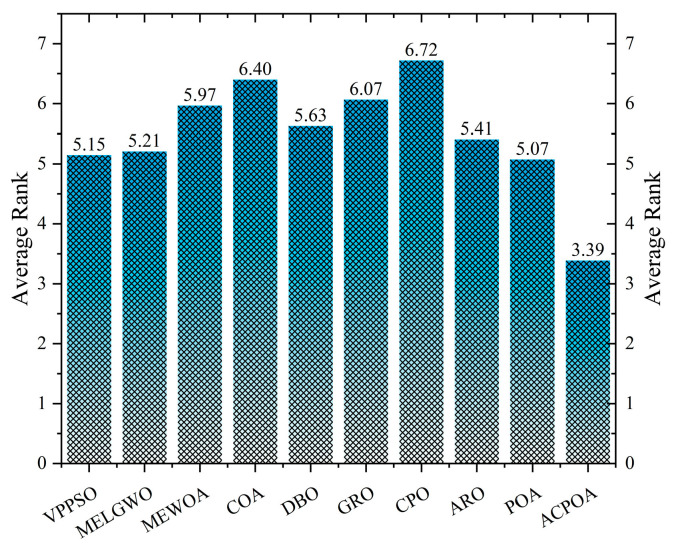
Average rank of SSIM in Otsu.

Table 10 reports the Structural Similarity Index (SSIM), which measures the consistency between the segmented and original images in terms of luminance, contrast, and structure. Values closer to 1 indicate better structural preservation. ACPOA shows a significant advantage on this metric. For example, in the 8-level thresholding of the face image, ACPOA achieves a mean SSIM of 0.8576, notably higher than POA’s 0.8416, MELGWO’s 0.8386, and MEWOA’s 0.8172, indicating better maintenance of the overall image structure. In the 6-level thresholding of the bank image, ACPOA’s SSIM is 0.8152, outperforming POA’s 0.8103 and COA’s 0.8041. As illustrated in Figure 16, ACPOA ranks first with an average rank of 3.39, far ahead of standard POA’s 5.07. This superiority is attributed to the elite pool mutation strategy, which integrates optimal individual information to guide the algorithm’s focus on threshold optimization in structurally critical regions. Additionally, ACPOA’s SSIM values remain stable across all threshold levels, with standard deviations generally lower than those of other algorithms. This reflects its effective balance between exploration and exploitation, preventing structural information loss caused by premature convergence.

Collectively, these results consistently demonstrate that POA outperforms comparative algorithms across multilevel thresholding image segmentation tasks. Its performance advantage arises from the synergistic effect of three key improvement strategies: the elite pool mutation strategy enhances the optimization accuracy of the Otsu objective function by guiding with optimal individuals; the adaptive cooperative mechanism improves search efficiency in high-dimensional threshold spaces through specialized subgroup division and global information sharing, resulting in better PSNR, FSIM, and SSIM scores; and the hybrid boundary handling strategy reduces ineffective fluctuations in threshold search, leading to smaller standard deviations and greater algorithm stability.

Whether dealing with simple images (such as bank) or complex ones (such as baboon), and whether performing low-level (2-level) or high-level (8-level) thresholding, ACPOA consistently produces superior results, fully validating its practicality and superiority in multilevel image segmentation.

## 5. Summary and Prospect

The proposed ACPOA algorithm achieves comprehensive performance improvements through three core enhancement strategies. First, the elite pool mutation strategy constructs a guidance mechanism based on the top three individuals with the best fitness values. This not only broadens the scope of global exploration but also enhances local exploitation precision, effectively preventing premature loss of population diversity and reducing the risk of getting trapped in local optima in complex multimodal functions. Second, the adaptive cooperative learning mechanism dynamically allocates subgroups and dimensions to specialized updates, enabling efficient search in high-dimensional spaces and addressing the adaptability shortcomings of standard algorithms in such problems. Third, the hybrid boundary handling technique significantly outperforms traditional truncation methods in optimization efficiency near boundary regions by preserving effective search information and maintaining population stability.

On both the CEC2017 and CEC2022 benchmark test sets, ACPOA achieves comprehensive performance improvements through the integration of its three core strategies. Specifically, on CEC2017 (30 dimensions) and CEC2022 (10 D/20 D), ACPOA attains average rankings of 1.47, 2.08, and 1.42, respectively, ranking first in all cases. Notably, ACPOA secures the top position on 16 out of 30 functions in CEC2017 and consistently maintains a top-2 ranking on CEC2022, significantly outperforming the original POA and competing algorithms such as VPPSO, MELGWO, and COA, whose average rankings range between 4.17–8.07. Statistical analyses, convergence curves, and rank distributions all validate ACPOA’s superiority in terms of convergence speed, optimization accuracy, and stability.

When applied to the multilevel threshold image segmentation task, ACPOA also demonstrates outstanding practical performance. For example, in the eight-level threshold segmentation of the “bank” image, ACPOA achieves a PSNR of 25.2639 dB (an improvement of 0.5 dB over POA), a FSIM of 0.9089 (+0.0054), and a SSIM of 0.8556 (+0.0063). Across five benchmark images and four threshold levels, ACPOA surpasses competing algorithms in PSNR, FSIM, and SSIM in 80% of the scenarios, fully demonstrating the synergistic effectiveness of its three enhancement strategies and laying a solid foundation for subsequent engineering applications.

Future research can further advance along the following directions: (1) designing an adaptive mechanism to dynamically adjust the elite pool size and update probability according to different types of optimization problems (e.g., unimodal/multimodal, low/high dimensional), thereby enabling the elite pool mutation strategy to flexibly balance exploration and exploitation; (2) extending the adaptive cooperative learning mechanism to broader applications, such as developing subgroup-based cooperative Pareto optimal search strategies for multi-objective optimization, enhancing the algorithm’s capability to handle conflicting objectives; (3) tailoring the hybrid boundary handling technique to specific real-world problems—such as the boundary ambiguity in medical images or energy constraints in wireless sensor networks—to improve practical applicability.

Additionally, integrating ACPOA with deep learning methods could be explored, for example by leveraging neural networks to predict fitness trends of elite pool individuals, further accelerating convergence and broadening the algorithm’s applicability and performance advantages across diverse domains.

## Figures and Tables

**Table 1:** Compare algorithm parameter settings.

Algorithms	Name of the Parameter	Value of the Parameter
VPPSO	$\alpha ,N_{1},N_{2}$	0.3, 0.15, 0.15
IAGWO	a	$\left[0,2\right]$
MEWOA	b,r,l,α	1, [0, 1], [−1, 1], [0, 2]
COA	$C_{1},C_{2},\mu ,\sigma$	0.2, 3, 25, 3
DBO	$P_{percent}$	0.2
GRO	$r_{1},r_{2},r_{3},m$	$\left[0,1\right],\left[0,1\right],\left[0,1\right],\left[0,1\right],$
CPO	$\alpha ,N_{min},T_{f},T$	0.1, 80, 0.5, 2
ARO	$n_{1},n_{2}$	N(0, 1), N(0, 1)
POA	I,R	{1, 2}, 0.2
ACPOA	I,R,P	{1, 2}, 0.2, [0, 1]

**Table 2:** Results obtained by different algorithms on CEC2017 test suite (dim = 30).

F~	Metric	VPPSO	MELGWO	MEWOA	COA	DBO	GRO	CPO	ARO	POA	ACPOA
F1	Mean	1.1375 × 10^7^	1.8308 × 10^9^	5.7291 × 10^9^	8.6894 × 10^8^	3.1419 × 10^8^	1.0261 × 10^8^	7.6019 × 10^5^	3.1496 × 10^7^	1.6176 × 10^10^	**3.9708 × 10^3^**
	Std	3.4628 × 10^7^	1.5588 × 10^9^	2.6310 × 10^9^	8.1337 × 10^8^	2.6897 × 10^8^	6.9240 × 10^7^	6.0890 × 10^5^	2.4351 × 10^7^	5.0407 × 10^9^	**4.6275 × 10^3^**
F2	Mean	1.2711 × 10^23^	3.4443 × 10^31^	6.6795 × 10^33^	2.2143 × 10^27^	1.4254 × 10^33^	6.7416 × 10^24^	**5.8416 × 10^20^**	7.6964 × 10^22^	1.2596 × 10^33^	9.8896 × 10^22^
	Std	5.3032 × 10^23^	1.3276 × 10^32^	2.5667 × 10^34^	8.4364 × 10^27^	6.6217 × 10^33^	1.4303 × 10^25^	**2.9370 × 10^21^**	2.7150 × 10^23^	3.7946 × 10^33^	5.4168 × 10^23^
F3	Mean	4.9827 × 10^4^	4.3420 × 10^4^	7.6186 × 10^4^	1.2071 × 10^5^	8.9042 × 10^4^	6.4340 × 10^4^	6.5215 × 10^4^	5.8754 × 10^4^	4.2778 × 10^4^	**3.5586 × 10^4^**
	Std	1.2072 × 10^4^	9.8805 × 10^3^	9.7287 × 10^3^	3.7089 × 10^4^	1.4271 × 10^4^	1.2942 × 10^4^	1.1539 × 10^4^	9.9056 × 10^3^	**8.5261 × 10^3^**	9.9549 × 10^3^
F4	Mean	5.3082 × 10^2^	6.2890 × 10^2^	9.0888 × 10^2^	6.0387 × 10^2^	6.9545 × 10^2^	5.5125 × 10^2^	5.2024 × 10^2^	5.4133 × 10^2^	2.4250 × 10^3^	**4.8022 × 10^2^**
	Std	3.7175 × 10^1^	1.2765 × 10^2^	2.5649 × 10^2^	8.7124 × 10^1^	1.6924 × 10^2^	2.4724 × 10^1^	**1.5100 × 10^1^**	2.8898 × 10^1^	1.3609 × 10^3^	2.9142 × 10^1^
F5	Mean	6.5220 × 10^2^	6.6833 × 10^2^	8.0535 × 10^2^	7.4572 × 10^2^	7.4933 × 10^2^	6.0438 × 10^2^	6.9201 × 10^2^	6.3877 × 10^2^	7.6623 × 10^2^	**5.9121 × 10^2^**
	Std	2.7897 × 10^1^	3.6359 × 10^1^	4.1191 × 10^1^	6.2452 × 10^1^	4.3479 × 10^1^	1.9339 × 10^1^	**1.3670 × 10^1^**	3.4135 × 10^1^	3.6758 × 10^1^	1.6589 × 10^1^
F6	Mean	6.3577 × 10^2^	6.4762 × 10^2^	6.6788 × 10^2^	6.5519 × 10^2^	6.4706 × 10^2^	6.0683 × 10^2^	6.0191 × 10^2^	6.1521 × 10^2^	6.6142 × 10^2^	**6.0018 × 10^2^**
	Std	1.1280 × 10^1^	1.0425 × 10^1^	8.6949 × 10^0^	1.0440 × 10^1^	1.1190 × 10^1^	2.0843 × 10^0^	1.0067 × 10^0^	7.4614 × 10^0^	6.0952 × 10^0^	**2.1633 × 10^−1^**
F7	Mean	9.4449 × 10^2^	1.0007 × 10^3^	1.2296 × 10^3^	1.2441 × 10^3^	1.0351 × 10^3^	8.5168 × 10^2^	9.4051 × 10^2^	9.5214 × 10^2^	1.2676 × 10^3^	**8.2402 × 10^2^**
	Std	6.0901 × 10^1^	5.4809 × 10^1^	1.0337 × 10^2^	1.0238 × 10^2^	9.5025 × 10^1^	**2.3671 × 10^1^**	2.4305 × 10^1^	8.0706 × 10^1^	5.7617 × 10^1^	2.5279 × 10^1^
F8	Mean	9.2337 × 10^2^	9.4264 × 10^2^	1.0340 × 10^3^	9.8202 × 10^2^	1.0328 × 10^3^	9.0508 × 10^2^	9.8309 × 10^2^	9.0285 × 10^2^	9.9329 × 10^2^	**8.9669 × 10^2^**
	Std	2.9430 × 10^1^	3.0132 × 10^1^	3.1492 × 10^1^	3.2143 × 10^1^	6.2159 × 10^1^	1.9836 × 10^1^	**1.5220 × 10^1^**	2.5415 × 10^1^	2.2220 × 10^1^	1.7811 × 10^1^
F9	Mean	3.6677 × 10^3^	4.0001 × 10^3^	8.1401 × 10^3^	7.5663 × 10^3^	6.9243 × 10^3^	**1.2713 × 10^3^**	1.3021 × 10^3^	2.8463 × 10^3^	5.7281 × 10^3^	1.4125 × 10^3^
	Std	1.2226 × 10^3^	7.8868 × 10^2^	1.3441 × 10^3^	1.9048 × 10^3^	1.7899 × 10^3^	**2.2074 × 10^2^**	3.2902 × 10^2^	8.6433 × 10^2^	7.4255 × 10^2^	4.5988 × 10^2^
F10	Mean	4.9128 × 10^3^	5.0444 × 10^3^	7.2141 × 10^3^	6.1829 × 10^3^	6.8688 × 10^3^	5.8291 × 10^3^	7.5081 × 10^3^	4.4758 × 10^3^	5.3002 × 10^3^	**4.2705 × 10^3^**
	Std	7.4816 × 10^2^	5.2892 × 10^2^	7.7563 × 10^2^	8.8919 × 10^2^	1.1659 × 10^3^	4.7016 × 10^2^	**3.8199 × 10^2^**	5.5755 × 10^2^	4.4992 × 10^2^	5.6168 × 10^2^
F11	Mean	1.4462 × 10^3^	1.4731 × 10^3^	3.2492 × 10^3^	1.7790 × 10^3^	1.8311 × 10^3^	1.3434 × 10^3^	1.2784 × 10^3^	1.3483 × 10^3^	2.3659 × 10^3^	**1.2021 × 10^3^**
	Std	1.6075 × 10^2^	4.0029 × 10^2^	1.2634 × 10^3^	4.8034 × 10^2^	5.4897 × 10^2^	7.3209 × 10^1^	2.7920 × 10^1^	1.1555 × 10^2^	1.1036 × 10^3^	**2.6893 × 10^1^**
F12	Mean	2.9636 × 10^7^	4.5530 × 10^7^	3.4326 × 10^8^	1.2786 × 10^7^	6.6984 × 10^7^	4.3046 × 10^6^	**1.2139 × 10^6^**	3.7488 × 10^6^	1.3619 × 10^9^	1.3642 × 10^6^
	Std	2.5541 × 10^7^	4.8988 × 10^7^	3.1841 × 10^8^	8.0430 × 10^6^	8.1928 × 10^7^	3.4547 × 10^6^	**7.1367 × 10^5^**	2.5990 × 10^6^	1.4644 × 10^9^	1.1044 × 10^6^
F13	Mean	9.3463 × 10^4^	1.5714 × 10^5^	2.0549 × 10^7^	3.3740 × 10^5^	5.0478 × 10^6^	1.0275 × 10^5^	2.0402 × 10^4^	1.7617 × 10^4^	1.4489 × 10^8^	**9.4868 × 10^3^**
	Std	5.3504 × 10^4^	4.0772 × 10^5^	3.6758 × 10^7^	6.6836 × 10^5^	7.5956 × 10^6^	1.4033 × 10^5^	1.1504 × 10^4^	1.4142 × 10^4^	4.6875 × 10^8^	**8.6454 × 10^3^**
F14	Mean	1.1363 × 10^5^	2.3274 × 10^5^	8.3826 × 10^5^	5.3779 × 10^5^	2.8492 × 10^5^	3.0620 × 10^4^	**2.3852 × 10^3^**	9.2270 × 10^4^	2.9205 × 10^4^	8.9124 × 10^4^
	Std	1.3570 × 10^5^	2.9271 × 10^5^	8.0865 × 10^5^	9.9421 × 10^5^	3.3388 × 10^5^	3.2617 × 10^4^	**1.3412 × 10^3^**	1.5947 × 10^5^	3.8543 × 10^4^	1.0446 × 10^5^
F15	Mean	3.8031 × 10^4^	2.3062 × 10^4^	4.6878 × 10^6^	2.8996 × 10^4^	7.0496 × 10^4^	2.5453 × 10^4^	5.1066 × 10^3^	4.5295 × 10^3^	4.5049 × 10^4^	**1.9796 × 10^3^**
	Std	1.9962 × 10^4^	1.3341 × 10^4^	1.3037 × 10^7^	2.9043 × 10^4^	7.5036 × 10^4^	1.6858 × 10^4^	3.7134 × 10^3^	2.6816 × 10^3^	3.1998 × 10^4^	**6.7979 × 10^2^**
F16	Mean	2.9233 × 10^3^	2.8686 × 10^3^	3.5036 × 10^3^	2.9869 × 10^3^	3.5284 × 10^3^	2.5235 × 10^3^	3.0865 × 10^3^	2.6728 × 10^3^	3.0648 × 10^3^	**2.3041 × 10^3^**
	Std	4.2665 × 10^2^	3.4690 × 10^2^	4.0548 × 10^2^	3.8779 × 10^2^	4.1437 × 10^2^	1.9900 × 10^2^	1.8006 × 10^2^	2.5857 × 10^2^	2.9187 × 10^2^	**1.6933 × 10^2^**
F17	Mean	2.2173 × 10^3^	2.2770 × 10^3^	2.4988 × 10^3^	2.2903 × 10^3^	2.6191 × 10^3^	**1.9040 × 10^3^**	2.0582 × 10^3^	2.1663 × 10^3^	2.2947 × 10^3^	1.9909 × 10^3^
	Std	1.6317 × 10^2^	2.4677 × 10^2^	2.3346 × 10^2^	2.0758 × 10^2^	2.5341 × 10^2^	**9.7707 × 10^1^**	1.3675 × 10^2^	2.1816 × 10^2^	2.2626 × 10^2^	1.5693 × 10^2^
F18	Mean	1.3513 × 10^6^	1.1908 × 10^6^	6.7675 × 10^6^	3.0641 × 10^6^	4.7491 × 10^6^	4.6233 × 10^5^	**1.5521 × 10^5^**	3.9553 × 10^5^	3.4603 × 10^5^	2.0732 × 10^5^
	Std	1.7307 × 10^6^	1.1436 × 10^6^	7.0456 × 10^6^	3.5154 × 10^6^	5.8993 × 10^6^	3.5555 × 10^5^	**1.4850 × 10^5^**	4.2848 × 10^5^	3.8950 × 10^5^	2.1897 × 10^5^
F19	Mean	2.0797 × 10^6^	2.7473 × 10^5^	5.0617 × 10^6^	2.1012 × 10^4^	5.7832 × 10^6^	3.2846 × 10^4^	5.8061 × 10^3^	9.6496 × 10^3^	1.1777 × 10^6^	**3.8020 × 10^3^**
	Std	1.3157 × 10^6^	1.0500 × 10^6^	6.8168 × 10^6^	2.2412 × 10^4^	1.1055 × 10^7^	5.6650 × 10^4^	3.6581 × 10^3^	6.2807 × 10^3^	1.1486 × 10^6^	**2.5795 × 10^3^**
F20	Mean	2.4563 × 10^3^	2.6103 × 10^3^	2.7958 × 10^3^	2.7211 × 10^3^	2.6765 × 10^3^	2.3393 × 10^3^	2.4494 × 10^3^	2.4309 × 10^3^	2.4947 × 10^3^	**2.2506 × 10^3^**
	Std	1.8709 × 10^2^	1.7798 × 10^2^	1.9587 × 10^2^	2.4077 × 10^2^	1.8525 × 10^2^	**8.8035 × 10^1^**	1.2807 × 10^2^	1.7563 × 10^2^	1.3419 × 10^2^	1.1985 × 10^2^
F21	Mean	2.4318 × 10^3^	2.4564 × 10^3^	2.5681 × 10^3^	2.4696 × 10^3^	2.5673 × 10^3^	2.3989 × 10^3^	2.4850 × 10^3^	2.4107 × 10^3^	2.5529 × 10^3^	**2.3926 × 10^3^**
	Std	3.6844 × 10^1^	3.3051 × 10^1^	3.9999 × 10^1^	3.7966 × 10^1^	4.3237 × 10^1^	1.9409 × 10^1^	**1.4974 × 10^1^**	2.9969 × 10^1^	4.3677 × 10^1^	1.9150 × 10^1^
F22	Mean	3.7881 × 10^3^	5.1707 × 10^3^	6.5027 × 10^3^	4.0338 × 10^3^	5.2794 × 10^3^	2.3565 × 10^3^	**2.3098 × 10^3^**	2.3472 × 10^3^	5.9579 × 10^3^	3.8288 × 10^3^
	Std	2.0541 × 10^3^	1.8701 × 10^3^	2.7016 × 10^3^	2.4042 × 10^3^	2.3332 × 10^3^	1.7858 × 10^1^	**2.5448 × 10^0^**	4.5840 × 10^1^	1.6821 × 10^3^	1.5900 × 10^3^
F23	Mean	2.8124 × 10^3^	2.8197 × 10^3^	2.9730 × 10^3^	2.8643 × 10^3^	2.9853 × 10^3^	2.7521 × 10^3^	2.8459 × 10^3^	2.7860 × 10^3^	3.0295 × 10^3^	**2.7482 × 10^3^**
	Std	3.5110 × 10^1^	4.4436 × 10^1^	7.0886 × 10^1^	6.5980 × 10^1^	7.3775 × 10^1^	**1.7045 × 10^1^**	1.9810 × 10^1^	3.8880 × 10^1^	7.2357 × 10^1^	1.9297 × 10^1^
F24	Mean	2.9629 × 10^3^	2.9717 × 10^3^	3.1026 × 10^3^	3.0250 × 10^3^	3.1508 × 10^3^	**2.9169 × 10^3^**	3.0174 × 10^3^	2.9578 × 10^3^	3.1927 × 10^3^	2.9552 × 10^3^
	Std	3.0418 × 10^1^	5.0854 × 10^1^	5.4099 × 10^1^	8.9759 × 10^1^	8.5100 × 10^1^	**1.5911 × 10^1^**	2.0416 × 10^1^	3.7326 × 10^1^	7.4481 × 10^1^	3.7275 × 10^1^
F25	Mean	2.9537 × 10^3^	2.9894 × 10^3^	3.1396 × 10^3^	2.9660 × 10^3^	2.9781 × 10^3^	2.9344 × 10^3^	2.9150 × 10^3^	2.9704 × 10^3^	3.2801 × 10^3^	**2.8925 × 10^3^**
	Std	2.3983 × 10^1^	5.1357 × 10^1^	9.5714 × 10^1^	3.0666 × 10^1^	1.4119 × 10^2^	2.0018 × 10^1^	1.6907 × 10^1^	2.4124 × 10^1^	2.2383 × 10^2^	**1.2564 × 10^1^**
F26	Mean	4.8078 × 10^3^	6.1398 × 10^3^	6.7816 × 10^3^	6.0131 × 10^3^	6.9286 × 10^3^	**4.2501 × 10^3^**	4.9561 × 10^3^	5.1848 × 10^3^	7.2540 × 10^3^	4.5868 × 10^3^
	Std	1.2737 × 10^3^	7.6364 × 10^2^	1.6242 × 10^3^	1.8671 × 10^3^	1.0119 × 10^3^	**6.6665 × 10^2^**	1.2473 × 10^3^	9.8753 × 10^2^	1.4921 × 10^3^	6.7159 × 10^2^
F27	Mean	3.2980 × 10^3^	3.2939 × 10^3^	3.3729 × 10^3^	3.2864 × 10^3^	3.3443 × 10^3^	3.2621 × 10^3^	3.2746 × 10^3^	3.2790 × 10^3^	3.3929 × 10^3^	**3.2244 × 10^3^**
	Std	4.4947 × 10^1^	4.7424 × 10^1^	9.8957 × 10^1^	4.8187 × 10^1^	7.8180 × 10^1^	1.4906 × 10^1^	**1.2546 × 10^1^**	2.8122 × 10^1^	9.9678 × 10^1^	1.3185 × 10^1^
F28	Mean	3.3128 × 10^3^	3.4574 × 10^3^	3.5863 × 10^3^	3.3904 × 10^3^	3.6925 × 10^3^	3.3070 × 10^3^	3.2864 × 10^3^	3.3538 × 10^3^	4.0754 × 10^3^	**3.2095 × 10^3^**
	Std	3.0797 × 10^1^	1.3534 × 10^2^	1.0354 × 10^2^	1.0071 × 10^2^	7.2494 × 10^2^	2.3882 × 10^1^	**1.8118 × 10^1^**	4.5569 × 10^1^	4.6386 × 10^2^	3.2677 × 10^1^
F29	Mean	4.3276 × 10^3^	4.3871 × 10^3^	4.9068 × 10^3^	4.1970 × 10^3^	4.4064 × 10^3^	3.8027 × 10^3^	3.9421 × 10^3^	3.9766 × 10^3^	4.5631 × 10^3^	**3.6825 × 10^3^**
	Std	2.9691 × 10^2^	3.4271 × 10^2^	5.0121 × 10^2^	3.0088 × 10^2^	3.3855 × 10^2^	2.0339 × 10^2^	**1.3313 × 10^2^**	2.0729 × 10^2^	3.1988 × 10^2^	1.3905 × 10^2^
F30	Mean	7.5835 × 10^6^	3.8007 × 10^6^	4.2896 × 10^7^	8.4434 × 10^5^	1.8720 × 10^6^	4.5062 × 10^5^	1.0977 × 10^5^	8.5338 × 10^4^	1.2651 × 10^7^	**1.6616 × 10^4^**
	Std	4.8053 × 10^6^	3.2224 × 10^6^	3.5696 × 10^7^	8.6091 × 10^5^	2.5307 × 10^6^	3.4936 × 10^5^	5.1837 × 10^4^	1.0351 × 10^5^	1.0297 × 10^7^	**1.3243 × 10^4^**
Mean. Rank		4.87	6.03	9.30	6.80	8.03	3.13	3.73	3.57	8.07	**1.47**
Friedman		5	6	10	7	8	2	4	3	9	**1**

**Table 3:** Results obtained by different algorithms on CEC2022 test suite (dim = 10).

F~	Metric	VPPSO	MELGWO	MEWOA	COA	DBO	GRO	CPO	ARO	POA	ACPOA
F1	Mean	3.4657 × 10^2^	3.0429 × 10^2^	1.9358 × 10^3^	3.9057 × 10^3^	2.0106 × 10^3^	4.8105 × 10^2^	3.9349 × 10^2^	5.0408 × 10^2^	7.9300 × 10^2^	**3.0003 × 10^2^**
	Std	1.0999 × 10^2^	1.3128 × 10^1^	1.7070 × 10^3^	2.7774 × 10^3^	1.7332 × 10^3^	3.7233 × 10^2^	8.9270 × 10^1^	3.4324 × 10^2^	9.1688 × 10^2^	**8.3926 × 10^−2^**
F2	Mean	4.1114 × 10^2^	4.1037 × 10^2^	4.3779 × 10^2^	4.1615 × 10^2^	4.3807 × 10^2^	4.0268 × 10^2^	**4.0064 × 10^2^**	4.0507 × 10^2^	4.2054 × 10^2^	4.0118 × 10^2^
	Std	1.7082 × 10^1^	1.6896 × 10^1^	3.4958 × 10^1^	2.6395 × 10^1^	3.6601 × 10^1^	3.3673 × 10^0^	**1.4780 × 10^0^**	1.2343 × 10^1^	2.6738 × 10^1^	1.9302 × 10^0^
F3	Mean	6.0734 × 10^2^	6.0792 × 10^2^	6.2528 × 10^2^	6.0749 × 10^2^	6.1192 × 10^2^	6.0004 × 10^2^	6.0000 × 10^2^	6.0015 × 10^2^	6.2215 × 10^2^	**6.0000 × 10^2^**
	Std	6.1923 × 10^0^	7.6987 × 10^0^	1.0269 × 10^1^	9.6619 × 10^0^	7.4745 × 10^0^	2.2921 × 10^−2^	3.1990 × 10^−3^	2.8397 × 10^−1^	1.1809 × 10^1^	**1.2467 × 10^−3^**
F4	Mean	8.1767 × 10^2^	8.1575 × 10^2^	8.2971 × 10^2^	8.2968 × 10^2^	8.3440 × 10^2^	**8.0968 × 10^2^**	8.2145 × 10^2^	8.1669 × 10^2^	8.1901 × 10^2^	8.1775 × 10^2^
	Std	5.7903 × 10^0^	6.8940 × 10^0^	8.1023 × 10^0^	5.7547 × 10^0^	1.2651 × 10^1^	**2.8398 × 10^0^**	5.1475 × 10^0^	6.8492 × 10^0^	5.9016 × 10^0^	5.4649 × 10^0^
F5	Mean	9.1339 × 10^2^	9.7621 × 10^2^	1.1846 × 10^3^	1.0600 × 10^3^	1.0028 × 10^3^	9.0006 × 10^2^	**9.0000 × 10^2^**	9.1070 × 10^2^	1.0933 × 10^3^	9.0275 × 10^2^
	Std	2.1344 × 10^1^	9.0952 × 10^1^	2.0945 × 10^2^	2.0665 × 10^2^	1.2525 × 10^2^	1.1693 × 10^−1^	**6.5088 × 10^−4^**	1.9006 × 10^1^	1.1774 × 10^2^	5.3010 × 10^0^
F6	Mean	4.4722 × 10^3^	3.7368 × 10^3^	1.6579 × 10^4^	4.7699 × 10^3^	5.2819 × 10^3^	2.8071 × 10^3^	**1.8282 × 10^3^**	2.7588 × 10^3^	2.8474 × 10^3^	1.8409 × 10^3^
	Std	2.2332 × 10^3^	2.1388 × 10^3^	1.5632 × 10^4^	1.8412 × 10^3^	2.2808 × 10^3^	9.9904 × 10^2^	**1.3051 × 10^1^**	1.1952 × 10^3^	1.3497 × 10^3^	1.1364 × 10^2^
F7	Mean	2.0402 × 10^3^	2.0412 × 10^3^	2.0550 × 10^3^	2.0266 × 10^3^	2.0415 × 10^3^	2.0146 × 10^3^	2.0111 × 10^3^	2.0143 × 10^3^	2.0394 × 10^3^	**2.0017 × 10^3^**
	Std	1.3565 × 10^1^	2.2983 × 10^1^	2.1811 × 10^1^	2.3171 × 10^1^	2.3737 × 10^1^	8.4558 × 10^0^	5.3486 × 10^0^	9.4758 × 10^0^	1.5977 × 10^1^	**5.0237 × 10^0^**
F8	Mean	2.2250 × 10^3^	2.2285 × 10^3^	2.2303 × 10^3^	2.2253 × 10^3^	2.2378 × 10^3^	2.2211 × 10^3^	2.2175 × 10^3^	2.2191 × 10^3^	2.2222 × 10^3^	**2.2135 × 10^3^**
	Std	**4.5283 × 10^0^**	2.3298 × 10^1^	5.3590 × 10^0^	9.5938 × 10^0^	3.0783 × 10^1^	5.4061 × 10^0^	5.6260 × 10^0^	4.9851 × 10^0^	8.1068 × 10^0^	9.5202 × 10^0^
F9	Mean	2.5325 × 10^3^	2.5308 × 10^3^	2.5615 × 10^3^	2.5342 × 10^3^	2.5620 × 10^3^	2.5293 × 10^3^	2.5293 × 10^3^	2.5294 × 10^3^	2.5447 × 10^3^	**2.5293 × 10^3^**
	Std	4.8412 × 10^0^	7.0711 × 10^0^	4.0100 × 10^1^	2.6826 × 10^1^	4.9916 × 10^1^	1.9516 × 10^−2^	5.2841 × 10^−3^	4.4776 × 10^−1^	2.0188 × 10^1^	**5.6355 × 10^−10^**
F10	Mean	2.5452 × 10^3^	2.5907 × 10^3^	2.5304 × 10^3^	2.5757 × 10^3^	2.5437 × 10^3^	**2.5076 × 10^3^**	2.5157 × 10^3^	2.5156 × 10^3^	2.5398 × 10^3^	2.5076 × 10^3^
	Std	5.9916 × 10^1^	1.6575 × 10^2^	5.4505 × 10^1^	1.3449 × 10^2^	6.1479 × 10^1^	**2.7629 × 10^1^**	3.9638 × 10^1^	3.9109 × 10^1^	6.0838 × 10^1^	5.8674 × 10^1^
F11	Mean	2.7651 × 10^3^	2.7579 × 10^3^	2.7460 × 10^3^	2.7806 × 10^3^	2.8091 × 10^3^	**2.6049 × 10^3^**	2.6233 × 10^3^	2.6225 × 10^3^	2.7512 × 10^3^	2.6795 × 10^3^
	Std	1.7757 × 10^2^	1.7065 × 10^2^	1.1913 × 10^2^	1.7163 × 10^2^	2.0082 × 10^2^	**2.1820 × 10^1^**	8.9763 × 10^1^	5.2027 × 10^1^	1.4235 × 10^2^	1.1109 × 10^2^
F12	Mean	**2.8640 × 10^3^**	2.8658 × 10^3^	2.8650 × 10^3^	2.8697 × 10^3^	2.8766 × 10^3^	2.8646 × 10^3^	2.8653 × 10^3^	2.8679 × 10^3^	2.8723 × 10^3^	2.8646 × 10^3^
	Std	1.1965 × 10^0^	3.9116 × 10^0^	1.7109 × 10^0^	1.3017 × 10^1^	1.8227 × 10^1^	**7.1820 × 10^−1^**	7.5474 × 10^−1^	4.4122 × 10^0^	1.8535 × 10^1^	1.3917 × 10^0^
Mean. Rank		5.17	5.33	9.00	6.92	8.67	3.42	2.92	4.08	7.42	**2.08**
Friedman		5	6	10	7	9	3	2	4	8	**1**

**Table 4:** Results obtained by different algorithms on CEC2022 test suite (dim = 20).

F~	Metric	VPPSO	MELGWO	MEWOA	COA	DBO	GRO	CPO	ARO	POA	ACPOA
F1	Mean	6.2969 × 10^3^	5.6957 × 10^3^	1.6995 × 10^4^	4.8599 × 10^4^	3.8080 × 10^4^	1.1417 × 10^4^	1.2658 × 10^4^	1.4647 × 10^4^	8.9836 × 10^3^	**2.2912 × 10^3^**
	Std	2.7746 × 10^3^	2.7018 × 10^3^	4.4983 × 10^3^	1.3467 × 10^4^	1.1222 × 10^4^	3.5870 × 10^3^	3.2792 × 10^3^	3.9141 × 10^3^	2.8026 × 10^3^	**1.3883 × 10^3^**
F2	Mean	4.8107 × 10^2^	5.1462 × 10^2^	5.8912 × 10^2^	5.0038 × 10^2^	5.0458 × 10^2^	4.6271 × 10^2^	4.5915 × 10^2^	4.8339 × 10^2^	6.4422 × 10^2^	**4.4991 × 10^2^**
	Std	2.8896 × 10^1^	3.9989 × 10^1^	6.5907 × 10^1^	3.6146 × 10^1^	8.0071 × 10^1^	**1.0024 × 10^1^**	1.0142 × 10^1^	3.2460 × 10^1^	1.0716 × 10^2^	1.9784 × 10^1^
F3	Mean	6.3019 × 10^2^	6.2784 × 10^2^	6.5362 × 10^2^	6.3468 × 10^2^	6.3436 × 10^2^	6.0167 × 10^2^	6.0033 × 10^2^	6.0539 × 10^2^	6.5430 × 10^2^	**6.0001 × 10^2^**
	Std	1.1186 × 10^1^	1.1766 × 10^1^	1.1987 × 10^1^	1.7618 × 10^1^	1.0589 × 10^1^	5.1073 × 10^−1^	1.3581 × 10^−1^	4.3495 × 10^0^	9.3883 × 10^0^	**2.1218 × 10^−2^**
F4	Mean	8.5977 × 10^2^	8.6492 × 10^2^	9.1100 × 10^2^	8.8575 × 10^2^	9.1892 × 10^2^	**8.4402 × 10^2^**	9.0038 × 10^2^	8.5377 × 10^2^	8.8068 × 10^2^	8.6319 × 10^2^
	Std	1.5268 × 10^1^	1.5199 × 10^1^	1.5716 × 10^1^	1.5254 × 10^1^	2.4063 × 10^1^	**7.8358 × 10^0^**	1.3335 × 10^1^	1.7456 × 10^1^	1.2753 × 10^1^	1.9148 × 10^1^
F5	Mean	1.5191 × 10^3^	1.6320 × 10^3^	2.8787 × 10^3^	2.6005 × 10^3^	2.1874 × 10^3^	9.2266 × 10^2^	**9.2261 × 10^2^**	1.2945 × 10^3^	2.2097 × 10^3^	1.2438 × 10^3^
	Std	3.6595 × 10^2^	3.6887 × 10^2^	5.0999 × 10^2^	5.9181 × 10^2^	5.2628 × 10^2^	**1.2432 × 10^1^**	3.9376 × 10^1^	2.7261 × 10^2^	1.7774 × 10^2^	2.4311 × 10^2^
F6	Mean	4.7761 × 10^3^	6.4213 × 10^3^	9.5032 × 10^6^	8.6735 × 10^3^	1.4453 × 10^6^	7.3043 × 10^4^	2.9895 × 10^4^	3.3147 × 10^3^	1.0228 × 10^6^	**2.5973 × 10^3^**
	Std	3.4746 × 10^3^	5.7911 × 10^3^	1.1634 × 10^7^	7.0119 × 10^3^	4.7778 × 10^6^	1.2657 × 10^5^	2.8984 × 10^4^	1.5890 × 10^3^	1.8377 × 10^6^	**9.2865 × 10^2^**
F7	Mean	2.1021 × 10^3^	2.1284 × 10^3^	2.1466 × 10^3^	2.1606 × 10^3^	2.1469 × 10^3^	2.0507 × 10^3^	2.0629 × 10^3^	2.0632 × 10^3^	2.1151 × 10^3^	**2.0297 × 10^3^**
	Std	4.0420 × 10^1^	6.7217 × 10^1^	3.4365 × 10^1^	1.0040 × 10^2^	6.1833 × 10^1^	1.0990 × 10^1^	**9.8261 × 10^0^**	3.1257 × 10^1^	3.8444 × 10^1^	1.2374 × 10^1^
F8	Mean	2.2661 × 10^3^	2.2762 × 10^3^	2.2812 × 10^3^	2.2748 × 10^3^	2.3359 × 10^3^	2.2296 × 10^3^	2.2315 × 10^3^	2.2323 × 10^3^	2.2642 × 10^3^	**2.2220 × 10^3^**
	Std	6.4708 × 10^1^	5.8535 × 10^1^	6.3978 × 10^1^	5.3474 × 10^1^	9.5727 × 10^1^	2.2382 × 10^0^	1.8118 × 10^0^	3.0063 × 10^1^	5.3938 × 10^1^	**1.3001 × 10^0^**
F9	Mean	2.5098 × 10^3^	2.5006 × 10^3^	2.5600 × 10^3^	2.4822 × 10^3^	2.5094 × 10^3^	2.4835 × 10^3^	2.4819 × 10^3^	2.4865 × 10^3^	2.5495 × 10^3^	**2.4808 × 10^3^**
	Std	1.9572 × 10^1^	1.7239 × 10^1^	4.1348 × 10^1^	2.7193 × 10^0^	2.6095 × 10^1^	1.1662 × 10^0^	7.1576 × 10^−1^	3.4248 × 10^0^	3.1790 × 10^1^	**2.6827 × 10^−4^**
F10	Mean	3.0863 × 10^3^	3.9147 × 10^3^	3.6152 × 10^3^	3.7418 × 10^3^	3.4571 × 10^3^	2.5383 × 10^3^	2.5387 × 10^3^	2.6418 × 10^3^	3.5533 × 10^3^	**2.4802 × 10^3^**
	Std	9.1275 × 10^2^	6.4224 × 10^2^	1.3826 × 10^3^	1.4204 × 10^3^	1.0735 × 10^3^	**6.3597 × 10^1^**	7.7304 × 10^1^	2.0358 × 10^2^	1.0430 × 10^3^	8.7741 × 10^1^
F11	Mean	2.9600 × 10^3^	3.1076 × 10^3^	3.8688 × 10^3^	3.3376 × 10^3^	3.1834 × 10^3^	3.0163 × 10^3^	**2.9152 × 10^3^**	2.9490 × 10^3^	4.8660 × 10^3^	2.9200 × 10^3^
	Std	2.5409 × 10^2^	2.2789 × 10^2^	6.4080 × 10^2^	7.5569 × 10^2^	5.1257 × 10^2^	8.0592 × 10^1^	7.3399 × 10^1^	6.8034 × 10^1^	8.7819 × 10^2^	**4.0680 × 10^1^**
F12	Mean	2.9879 × 10^3^	2.9828 × 10^3^	3.0129 × 10^3^	2.9995 × 10^3^	3.0316 × 10^3^	2.9682 × 10^3^	2.9886 × 10^3^	2.9829 × 10^3^	3.0570 × 10^3^	**2.9520 × 10^3^**
	Std	4.6786 × 10^1^	2.7982 × 10^1^	4.3618 × 10^1^	3.7041 × 10^1^	4.5918 × 10^1^	1.1385 × 10^1^	**1.0707 × 10^1^**	2.0398 × 10^1^	6.2539 × 10^1^	1.0873 × 10^1^
Mean. Rank		4.42	5.75	9.00	7.25	7.67	3.25	4.17	3.92	8.17	**1.42**
Friedman		5	6	10	7	8	2	4	3	9	**1**

**Table 5:** Time complexity comparison of different algorithms.

Algorithm	Time Complexity	Complexity Analysis
VPPSO	O (T × N × dim)	The velocity pause mechanism adds a constant-time judgment operation (O (1)) for each particle, and the overall complexity is dominated by iteration (T), population size (N), Reverse Learning (N),and dimension (dim)
MELGWO	O (T × N × dim)	The adaptive strategy introduces a constant-time parameter adjustment (O (1)), and the hierarchy update of grey wolves is O (N) (sorting), so the overall complexity is O (T × (N × dim + N)) = O (T × N × dim)
MEWOA	O (T × N × dim)	The moulting behavior requires a constant-time boundary judgment (O (1)), and the foraging update is O (N × dim), so the overall complexity is O (T × N × dim)
COA	O (T × N × dim)	The moulting behavior requires a constant-time boundary judgment (O (1)), and the foraging update is O (N × dim), so the overall complexity is O (T × N × dim)
DBO	O (T × N × dim)	The stealing behavior adds a constant-time random selection (O (1)) for each individual, and the dominant term is still O (T × N × dim)
GRO	O (T × N × dim)	The prospecting direction update is O (N × dim), and the digging depth adjustment is O (1), so the complexity is O (T × N × dim)
CPO	O (T × N × dim)	The quill defense mechanism adds a constant-time distance calculation (O (1)), and the overall complexity is dominated by iteration and population-dimension update
ARO	O (T × N × dim)	The hiding behavior requires a constant-time position randomization (O (1)), and the foraging update is O (N×dim), so the complexity is O (T × N × dim)
POA	O (T × N × dim)	The two-phase update (exploration + exploitation) is O (N × dim) per iteration, and no additional high-complexity operations are introduced
ACPOA	O (T × N × dim)	1. Elite pool mutation: Selecting top 3 individuals requires O (N) sorting (constant-time for small N), so O (1); 2. Adaptive cooperative mechanism: Subgroup-dimension allocation uses roulette wheel selection (O(dim) per subgroup, S = min(4, dim) is constant), so O (dim) = O (1); 3. Hybrid boundary handling: Probabilistic repair is O (1) per individual. The dominant term is still O (T × N × dim), which is consistent with the baseline POA

**Table 6:** ACPOA results of multi-level threshold segmentation with Otsu as the objective function.

Image	Threshold = 2	Threshold = 4	Threshold = 6	Threshold = 8
baboon	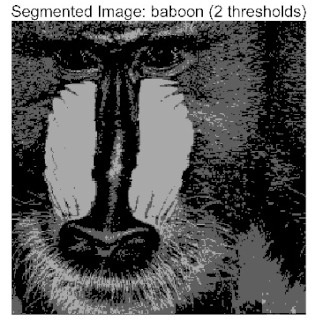	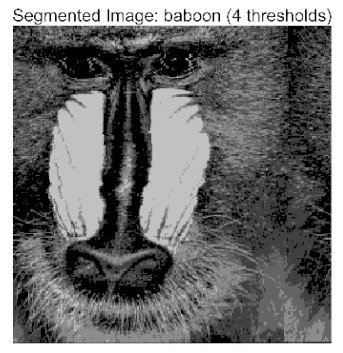	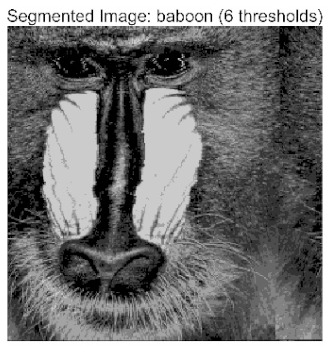	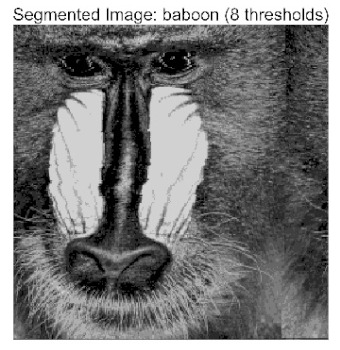
	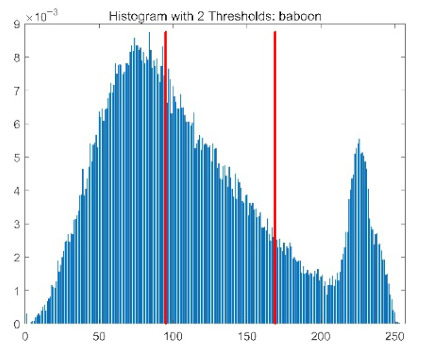	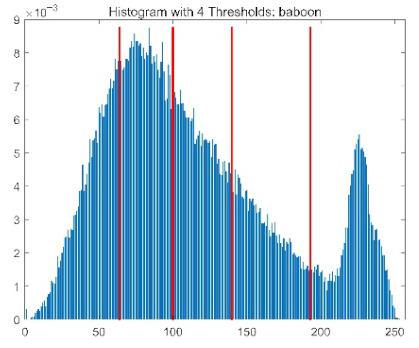	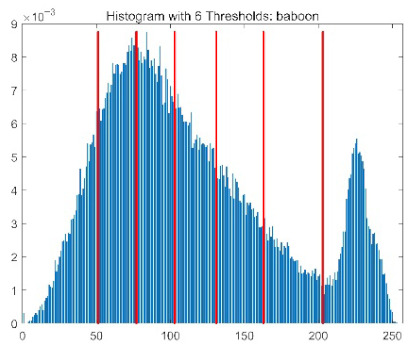	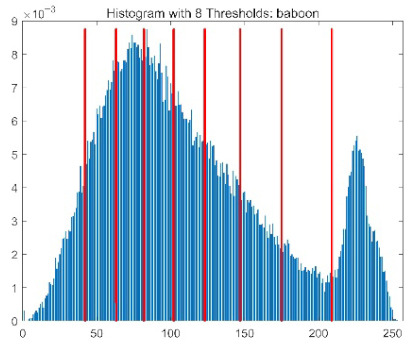
bank	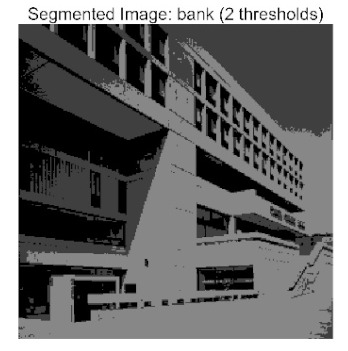	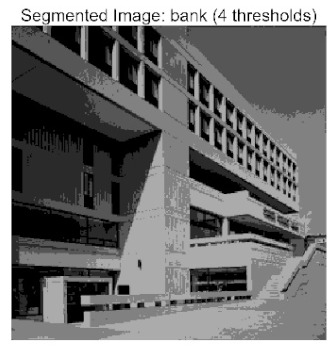	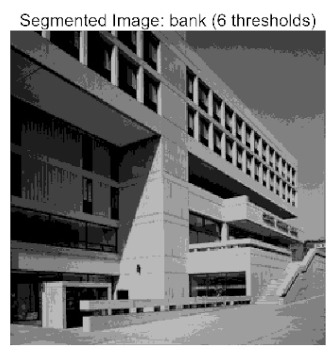	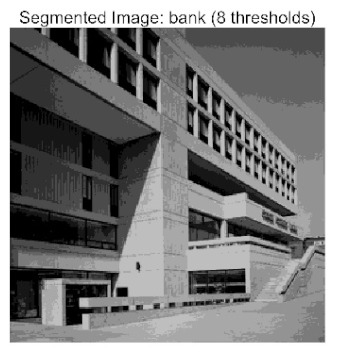
	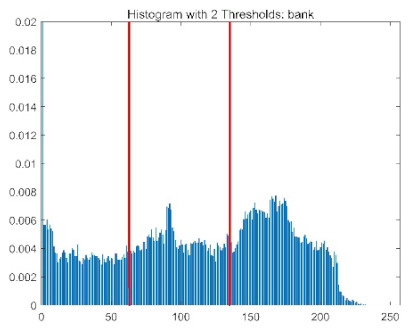	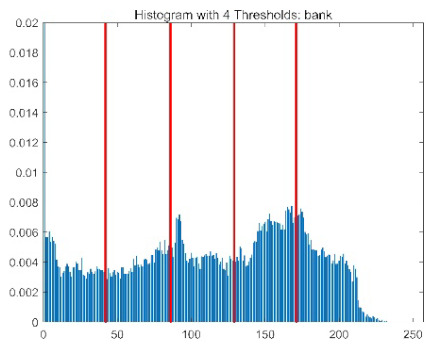	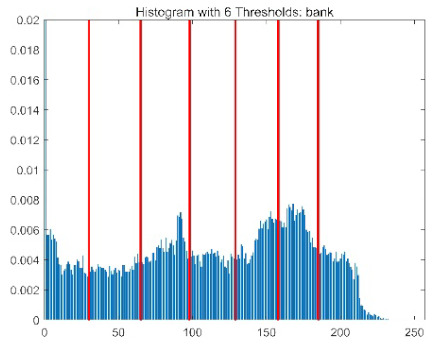	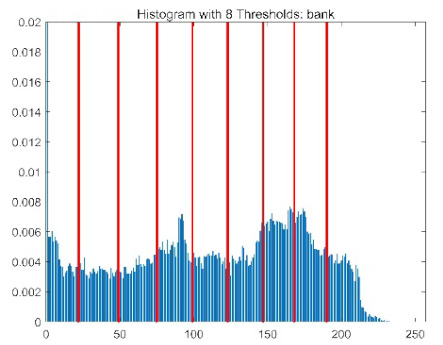
camera	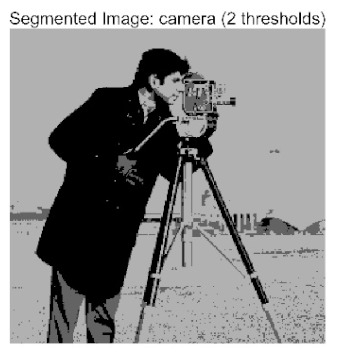	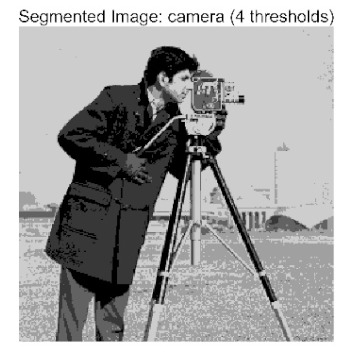	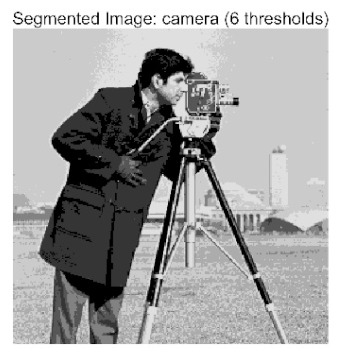	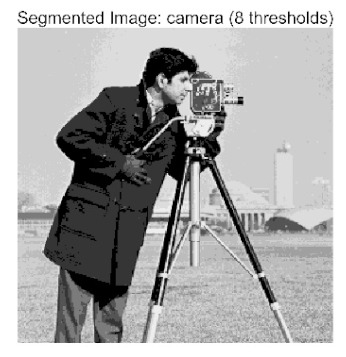
	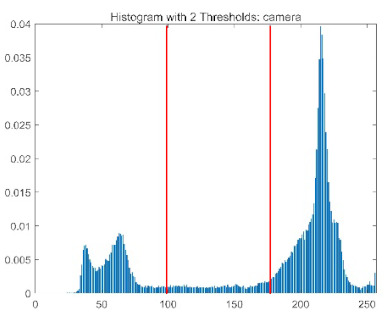	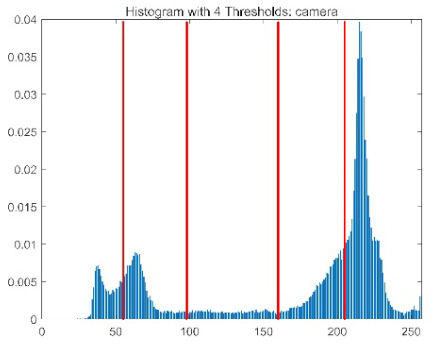	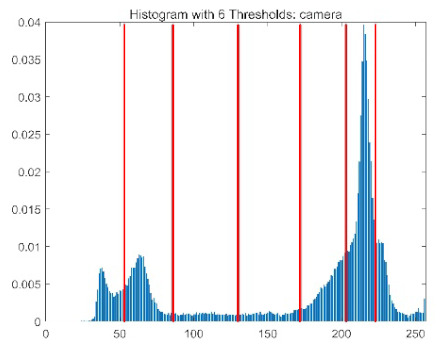	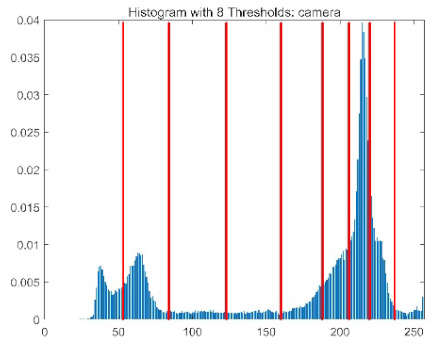
face	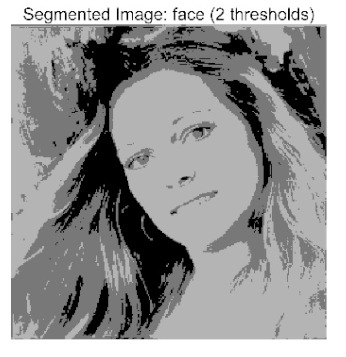	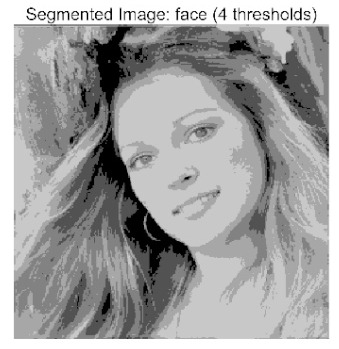	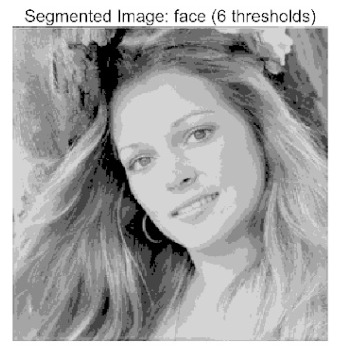	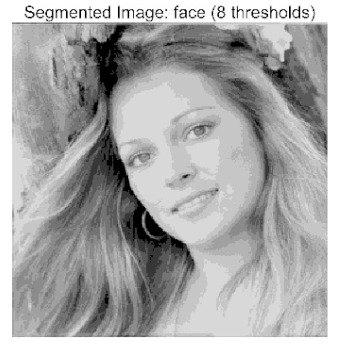
	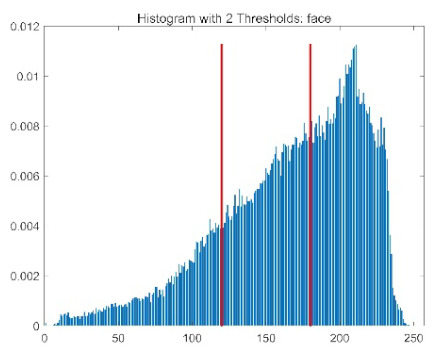	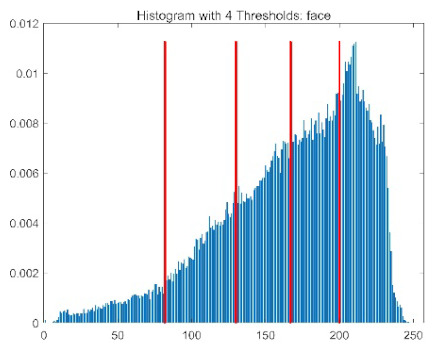	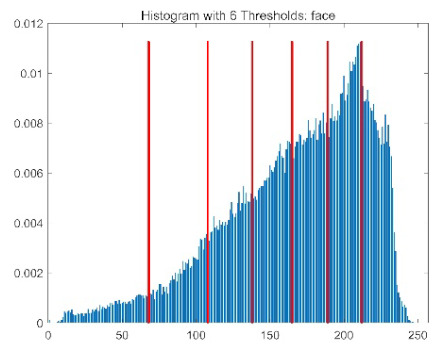	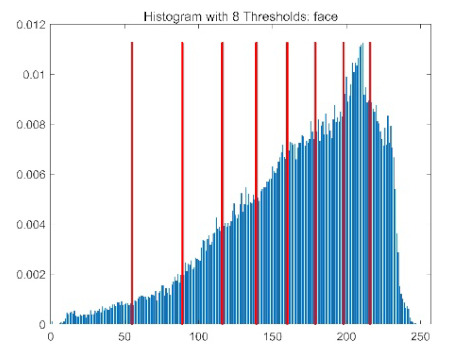
lena	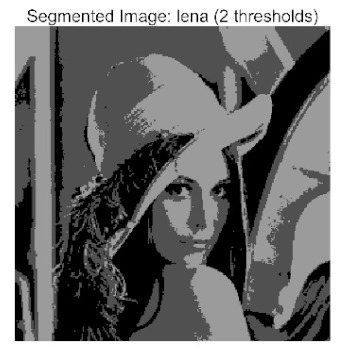	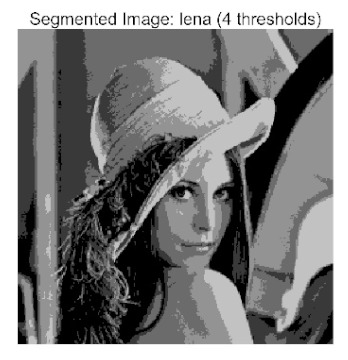	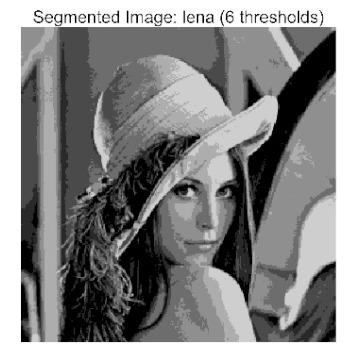	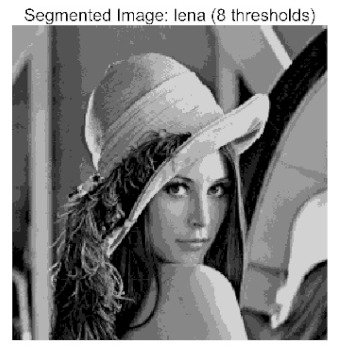
	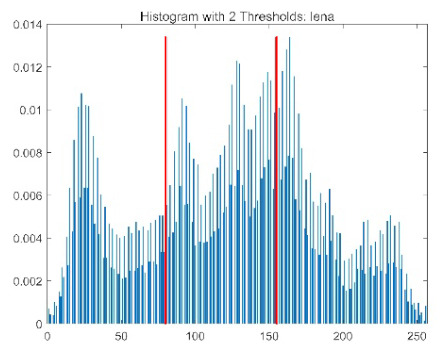	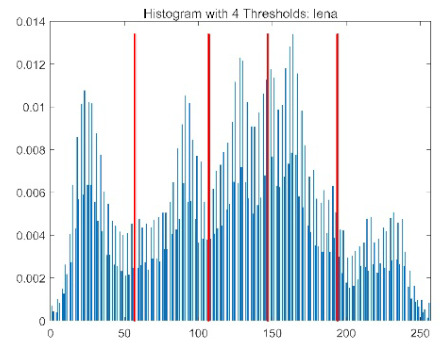	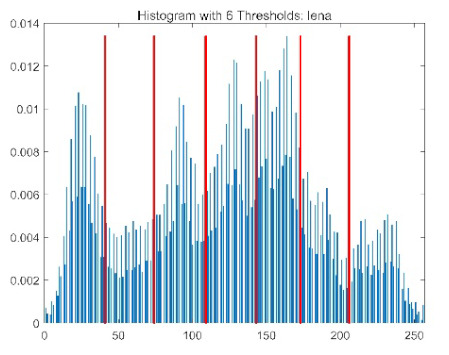	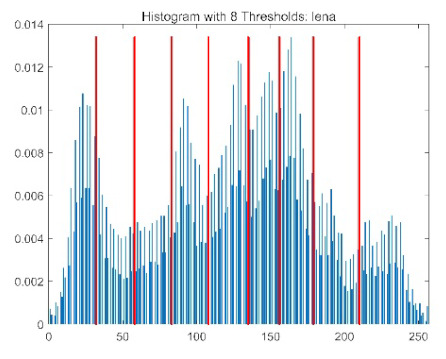

**Table 7:** Mean and Std of the optimal fitness values with Otsu as the objective function.

Image	Threshold	Metric	VPPSO	MELGWO	MEWOA	COA	DBO	GRO	CPO	ARO	POA	ACPOA
baboon	2	Mean	3.02 × 10^3^	3.02 × 10^3^	3.02 × 10^3^	3.02 × 10^3^	3.02 × 10^3^	3.02 × 10^3^	3.02 × 10^3^	3.02 × 10^3^	**3.02 × 10^3^**	**3.02 × 10^3^**
		Std	8.35 × 10^−3^	1.16 × 10^−1^	3.86 × 10^−1^	1.14 × 10^0^	3.48 × 10^−2^	5.19 × 10^−1^	8.03 × 10^−1^	1.23 × 10^−1^	**1.85 × 10^−12^**	**1.85 × 10^−12^**
	4	Mean	3.30 × 10^3^	3.30 × 10^3^	3.29 × 10^3^	3.29 × 10^3^	3.30 × 10^3^	3.30 × 10^3^	3.29 × 10^3^	3.30 × 10^3^	3.30 × 10^3^	**3.30 × 10^3^**
		Std	8.82 × 10^−1^	4.13 × 10^0^	4.01 × 10^0^	1.15 × 10^1^	2.44 × 10^0^	3.83 × 10^0^	5.80 × 10^0^	3.09 × 10^0^	1.19 × 10^0^	**1.50 × 10^−2^**
	6	Mean	3.37 × 10^3^	3.37 × 10^3^	3.36 × 10^3^	3.36 × 10^3^	3.36 × 10^3^	3.36 × 10^3^	3.36 × 10^3^	3.36 × 10^3^	3.37 × 10^3^	**3.37 × 10^3^**
		Std	3.35 × 10^0^	3.69 × 10^0^	1.05 × 10^1^	1.05 × 10^1^	5.16 × 10^0^	5.09 × 10^0^	4.53 × 10^0^	4.31 × 10^0^	2.32 × 10^0^	**1.03 × 10^−1^**
	8	Mean	3.40 × 10^3^	3.39 × 10^3^	3.38 × 10^3^	3.38 × 10^3^	3.39 × 10^3^	3.39 × 10^3^	3.39 × 10^3^	3.39 × 10^3^	3.40 × 10^3^	**3.40 × 10^3^**
		Std	3.14 × 10^0^	7.06 × 10^0^	1.03 × 10^1^	6.94 × 10^0^	5.14 × 10^0^	3.29 × 10^0^	3.73 × 10^0^	3.79 × 10^0^	2.12 × 10^0^	**1.70 × 10^−1^**
bank	2	Mean	**3.34 × 10^3^**	3.34 × 10^3^	3.34 × 10^3^	3.34 × 10^3^	3.34 × 10^3^	3.34 × 10^3^	3.34 × 10^3^	3.34 × 10^3^	**3.34 × 10^3^**	**3.34 × 10^3^**
		Std	**0.00 × 10^0^**	6.34 × 10^−1^	4.20 × 10^−1^	2.08 × 10^0^	3.50 × 10^−2^	1.99 × 10^−1^	5.75 × 10^−1^	2.06 × 10^−1^	**0.00 × 10^0^**	**0.00 × 10^0^**
	4	Mean	3.60 × 10^3^	3.60 × 10^3^	3.59 × 10^3^	3.59 × 10^3^	3.60 × 10^3^	3.60 × 10^3^	3.59 × 10^3^	3.60 × 10^3^	3.60 × 10^3^	**3.60 × 10^3^**
		Std	6.66 × 10^−1^	2.90 × 10^0^	1.35 × 10^1^	7.87 × 10^0^	2.84 × 10^0^	4.29 × 10^0^	3.47 × 10^0^	2.25 × 10^0^	9.81 × 10^−1^	**3.81 × 10^−2^**
	6	Mean	3.68 × 10^3^	3.67 × 10^3^	3.66 × 10^3^	3.67 × 10^3^	3.67 × 10^3^	3.67 × 10^3^	3.67 × 10^3^	3.67 × 10^3^	3.68 × 10^3^	**3.68 × 10^3^**
		Std	3.48 × 10^0^	6.42 × 10^0^	1.20 × 10^1^	9.13 × 10^0^	7.59 × 10^0^	5.03 × 10^0^	5.65 × 10^0^	3.18 × 10^0^	3.01 × 10^0^	**1.33 × 10^−1^**
	8	Mean	3.70 × 10^3^	3.70 × 10^3^	3.70 × 10^3^	3.70 × 10^3^	3.70 × 10^3^	3.70 × 10^3^	3.70 × 10^3^	3.70 × 10^3^	3.71 × 10^3^	**3.71 × 10^3^**
		Std	4.05 × 10^0^	7.51 × 10^0^	6.21 × 10^0^	8.53 × 10^0^	6.76 × 10^0^	4.20 × 10^0^	3.61 × 10^0^	2.97 × 10^0^	3.10 × 10^0^	**3.12 × 10^−1^**
camera	2	Mean	4.48 × 10^3^	4.48 × 10^3^	4.48 × 10^3^	4.48 × 10^3^	4.48 × 10^3^	**4.48 × 10^3^**	4.48 × 10^3^	4.48 × 10^3^	4.48 × 10^3^	**4.48 × 10^3^**
		Std	2.66 × 10^−2^	1.30 × 10^−1^	7.61 × 10^−2^	1.14 × 10^0^	1.99 × 10^−2^	**0.00 × 10^0^**	2.88 × 10^−1^	7.52 × 10^−2^	7.10 × 10^−2^	**0.00 × 10^0^**
	4	Mean	4.60 × 10^3^	4.60 × 10^3^	4.60 × 10^3^	4.60 × 10^3^	4.60 × 10^3^	4.60 × 10^3^	4.60 × 10^3^	4.60 × 10^3^	4.60 × 10^3^	**4.60 × 10^3^**
		Std	1.13 × 10^0^	1.54 × 10^0^	3.41 × 10^0^	2.19 × 10^0^	2.36 × 10^0^	2.02 × 10^0^	2.15 × 10^0^	1.62 × 10^0^	9.80 × 10^−1^	**1.34 × 10^−2^**
	6	Mean	4.65 × 10^3^	4.65 × 10^3^	4.64 × 10^3^	4.64 × 10^3^	4.64 × 10^3^	4.64 × 10^3^	4.64 × 10^3^	4.64 × 10^3^	4.65 × 10^3^	**4.65 × 10^3^**
		Std	4.49 × 10^0^	5.42 × 10^0^	6.58 × 10^0^	8.38 × 10^0^	5.78 × 10^0^	3.92 × 10^0^	4.71 × 10^0^	3.20 × 10^0^	2.72 × 10^0^	**7.40 × 10^−2^**
	8	Mean	4.67 × 10^3^	4.66 × 10^3^	4.66 × 10^3^	4.66 × 10^3^	4.66 × 10^3^	4.66 × 10^3^	4.66 × 10^3^	4.66 × 10^3^	4.66 × 10^3^	**4.67 × 10^3^**
		Std	2.85 × 10^0^	3.66 × 10^0^	5.40 × 10^0^	4.91 × 10^0^	4.75 × 10^0^	3.14 × 10^0^	2.73 × 10^0^	2.98 × 10^0^	2.43 × 10^0^	**4.05 × 10^−1^**
face	2	Mean	1.91 × 10^3^	1.91 × 10^3^	1.91 × 10^3^	1.91 × 10^3^	1.91 × 10^3^	1.91 × 10^3^	1.91 × 10^3^	1.91 × 10^3^	1.91 × 10^3^	**1.91 × 10^3^**
		Std	1.68 × 10^−1^	5.84 × 10^−1^	3.55 × 10^−1^	7.11 × 10^−1^	9.91 × 10^−2^	1.56 × 10^−1^	6.32 × 10^−1^	3.14 × 10^−1^	1.52 × 10^−2^	**6.94 × 10^−13^**
	4	Mean	2.12 × 10^3^	2.12 × 10^3^	2.12 × 10^3^	2.12 × 10^3^	2.12 × 10^3^	2.12 × 10^3^	2.11 × 10^3^	2.12 × 10^3^	2.12 × 10^3^	**2.12 × 10^3^**
		Std	6.95 × 10^−1^	3.51 × 10^0^	6.15 × 10^0^	7.79 × 10^0^	2.75 × 10^0^	3.25 × 10^0^	3.98 × 10^0^	2.52 × 10^0^	1.75 × 10^0^	**5.48 × 10^−2^**
	6	Mean	2.18 × 10^3^	2.18 × 10^3^	2.17 × 10^3^	2.17 × 10^3^	2.17 × 10^3^	2.17 × 10^3^	2.17 × 10^3^	2.18 × 10^3^	2.18 × 10^3^	**2.18 × 10^3^**
		Std	2.60 × 10^0^	4.89 × 10^0^	8.35 × 10^0^	7.89 × 10^0^	9.25 × 10^0^	3.79 × 10^0^	4.20 × 10^0^	3.29 × 10^0^	3.23 × 10^0^	**1.44 × 10^−1^**
	8	Mean	2.21 × 10^3^	2.20 × 10^3^	2.19 × 10^3^	2.20 × 10^3^	2.20 × 10^3^	2.20 × 10^3^	2.20 × 10^3^	2.20 × 10^3^	2.20 × 10^3^	**2.21 × 10^3^**
		Std	3.18 × 10^0^	4.26 × 10^0^	9.80 × 10^0^	7.21 × 10^0^	6.61 × 10^0^	3.41 × 10^0^	3.71 × 10^0^	2.64 × 10^0^	3.02 × 10^0^	**2.61 × 10^−1^**
lena	2	Mean	3.30 × 10^3^	3.30 × 10^3^	3.30 × 10^3^	3.30 × 10^3^	3.30 × 10^3^	3.30 × 10^3^	**3.30 × 10^3^**	3.30 × 10^3^	3.30 × 10^3^	3.30 × 10^3^
		Std	1.10 × 10^−1^	8.64 × 10^−1^	2.98 × 10^−1^	1.16 × 10^0^	2.08 × 10^−1^	2.86 × 10^−1^	**1.85 × 10^−12^**	3.65 × 10^−1^	7.88 × 10^−1^	1.85 × 10^−12^
	4	Mean	3.69 × 10^3^	3.68 × 10^3^	3.67 × 10^3^	3.68 × 10^3^	3.69 × 10^3^	3.68 × 10^3^	3.68 × 10^3^	3.68 × 10^3^	3.69 × 10^3^	**3.69 × 10^3^**
		Std	8.52 × 10^−1^	3.66 × 10^0^	1.56 × 10^1^	1.63 × 10^1^	1.13 × 10^0^	4.48 × 10^0^	4.94 × 10^0^	3.05 × 10^0^	8.54 × 10^−1^	**2.28 × 10^−2^**
	6	Mean	3.76 × 10^3^	3.76 × 10^3^	3.75 × 10^3^	3.75 × 10^3^	3.75 × 10^3^	3.75 × 10^3^	3.75 × 10^3^	3.76 × 10^3^	3.76 × 10^3^	**3.77 × 10^3^**
		Std	4.63 × 10^0^	6.52 × 10^0^	1.22 × 10^1^	1.29 × 10^1^	1.05 × 10^1^	5.26 × 10^0^	5.89 × 10^0^	4.02 × 10^0^	2.74 × 10^0^	**7.58 × 10^−2^**
	8	Mean	3.79 × 10^3^	3.79 × 10^3^	3.78 × 10^3^	3.78 × 10^3^	3.79 × 10^3^	3.79 × 10^3^	3.78 × 10^3^	3.79 × 10^3^	3.79 × 10^3^	**3.80 × 10^3^**
		Std	2.86 × 10^0^	5.84 × 10^0^	6.92 × 10^0^	5.82 × 10^0^	5.44 × 10^0^	4.83 × 10^0^	4.26 × 10^0^	2.39 × 10^0^	2.05 × 10^0^	**2.44 × 10^−1^**
Average rank			2.93	4.36	7.52	6.95	5.86	7.03	7.96	5.96	4.17	**2.25**
Rank			2	4	9	7	5	8	10	6	3	**1**

**Table 8:** Mean and Std of all test images for PSNR in Otsu.

Image	Threshold	Metric	VPPSO	MELGWO	MEWOA	COA	DBO	GRO	CPO	ARO	POA	ACPOA
baboon	2	Mean	**13.3365**	13.3290	13.3249	13.3267	13.3344	13.3200	13.3101	**13.3422**	13.3364	13.3364
		Std	7.73 × 10^−4^	3.38 × 10^−2^	5.42 × 10^−2^	5.79 × 10^−2^	1.17 × 10^−2^	5.23 × 10^−2^	8.01 × 10^−2^	3.88 × 10^−2^	**9.03 × 10^−15^**	**9.03 × 10^−15^**
	4	Mean	0.7202	0.7256	0.7277	17.9880	18.1284	18.0846	17.8792	18.1788	**18.2473**	18.1973
		Std	**7.92 × 10^−3^**	8.22 × 10^−3^	2.06 × 10^−2^	5.15 × 10^−1^	3.07 × 10^−1^	5.48 × 10^−1^	5.67 × 10^−1^	3.72 × 10^−1^	1.66 × 10^−1^	3.48 × 10^−2^
	6	Mean	21.2406	21.0986	21.0529	20.9320	20.6578	21.0330	20.7988	21.1048	21.3365	**21.3841**
		Std	5.05 × 10^−1^	5.77 × 10^−1^	9.22 × 10^−1^	8.76 × 10^−1^	7.55 × 10^−1^	6.08 × 10^−1^	7.65 × 10^−1^	5.21 × 10^−1^	4.31 × 10^−1^	**1.21 × 10^−1^**
	8	Mean	23.0891	23.0674	22.6065	22.4727	22.7880	22.9362	22.7874	23.1520	23.2291	**23.7296**
		Std	8.13 × 10^−1^	7.10 × 10^−1^	7.74 × 10^−1^	1.02 × 10^0^	6.94 × 10^−1^	7.77 × 10^−1^	7.45 × 10^−1^	4.39 × 10^−1^	6.86 × 10^−1^	**1.51 × 10^−1^**
bank	2	Mean	16.1237	16.1124	**16.1299**	16.1213	16.1230	16.1192	16.1282	16.1109	16.1237	16.1237
		Std	**1.08 × 10^−14^**	3.07 × 10^−2^	4.33 × 10^−2^	7.95 × 10^−2^	6.99 × 10^−3^	3.96 × 10^−2^	6.46 × 10^−2^	3.36 × 10^−2^	**1.08 × 10^−14^**	**1.08 × 10^−14^**
	4	Mean	20.1953	20.1507	19.9025	20.0087	20.1336	20.1161	20.0426	20.0276	20.1821	**20.2465**
		Std	7.94 × 10^−2^	1.27 × 10^−1^	3.50 × 10^−1^	2.25 × 10^−1^	1.13 × 10^−1^	1.64 × 10^−1^	1.94 × 10^−1^	1.67 × 10^−1^	9.39 × 10^−2^	**1.25 × 10^−2^**
	6	Mean	22.9213	22.8241	22.3843	22.3978	22.6451	22.5867	22.5365	22.6517	22.8934	**23.0945**
		Std	1.84 × 10^−1^	2.36 × 10^−1^	5.38 × 10^−1^	3.73 × 10^−1^	3.76 × 10^−1^	3.10 × 10^−1^	2.99 × 10^−1^	2.21 × 10^−1^	1.82 × 10^−1^	**2.99 × 10^−2^**
	8	Mean	24.7295	24.6335	24.1407	24.0948	24.2443	24.2816	24.2610	24.4456	24.7618	**25.2639**
		Std	3.15 × 10^−1^	5.40 × 10^−1^	4.30 × 10^−1^	6.28 × 10^−1^	4.71 × 10^−1^	3.66 × 10^−1^	2.87 × 10^−1^	2.66 × 10^−1^	3.08 × 10^−1^	**5.18 × 10^−2^**
camera	2	Mean	15.0255	15.0097	15.0406	14.9997	15.0432	15.0081	15.0181	15.0084	**15.0526**	**15.0526**
		Std	3.88 × 10^−2^	6.10 × 10^−2^	2.62 × 10^−2^	1.63 × 10^−1^	3.07 × 10^−2^	6.20 × 10^−2^	1.05 × 10^−1^	7.48 × 10^−2^	**0.00 × 10^0^**	**0.00 × 10^0^**
	4	Mean	18.7913	18.2094	18.3714	18.4111	18.3811	18.6016	18.5922	18.7677	19.5536	**19.8549**
		Std	8.56 × 10^−1^	6.30 × 10^−1^	1.03 × 10^0^	9.71 × 10^−1^	8.88 × 10^−1^	7.56 × 10^−1^	9.21 × 10^−1^	8.74 × 10^−1^	5.00 × 10^−1^	**3.70 × 10^−2^**
	6	Mean	21.2128	21.2606	20.7990	20.6149	21.4165	21.0631	21.1030	21.3045	21.5659	**21.8909**
		Std	8.96 × 10^−1^	8.21 × 10^−1^	1.17 × 10^0^	1.17 × 10^0^	7.76 × 10^−1^	8.61 × 10^−1^	1.07 × 10^0^	8.14 × 10^−1^	5.25 × 10^−1^	**5.73 × 10^−2^**
	8	Mean	22.8667	22.7975	22.5461	22.4843	22.5379	22.7340	22.6390	22.7709	22.7929	**22.9852**
		Std	5.64 × 10^−1^	7.86 × 10^−1^	1.08 × 10^0^	9.80 × 10^−1^	7.58 × 10^−1^	7.87 × 10^−1^	7.56 × 10^−1^	8.73 × 10^−1^	7.20 × 10^−1^	**1.72 × 10^−1^**
face	2	Mean	14.3031	14.2966	**14.3254**	14.3093	14.3079	14.2966	14.2850	14.2922	14.3202	14.3225
		Std	6.25 × 10^−2^	1.08 × 10^−1^	5.65 × 10^−2^	1.00 × 10^−1^	4.26 × 10^−2^	8.22 × 10^−2^	1.43 × 10^−1^	8.78 × 10^−2^	1.24 × 10^−2^	**1.81× 10^−15^**
	4	Mean	19.6645	19.6634	19.4970	19.5810	19.6764	19.5600	19.3900	19.6174	19.6275	**19.7572**
		Std	1.19 × 10^−1^	1.28 × 10^−1^	2.90 × 10^−1^	2.94 × 10^−1^	1.69 × 10^−1^	2.28 × 10^−1^	3.61 × 10^−1^	1.93 × 10^−1^	1.72 × 10^−1^	**2.59 × 10^−2^**
	6	Mean	22.3993	22.3944	21.8273	22.0686	22.0867	22.0759	22.0064	22.1338	22.4368	**22.5815**
		Std	2.76 × 10^−1^	3.13 × 10^−1^	5.47 × 10^−1^	4.27 × 10^−1^	4.88 × 10^−1^	4.05 × 10^−1^	4.08 × 10^−1^	3.71 × 10^−1^	2.85 × 10^−1^	**5.48 × 10^−2^**
	8	Mean	24.3443	24.1981	23.5769	23.8893	23.8315	23.7570	23.8162	24.1000	24.3424	**24.9131**
		Std	4.00 × 10^−1^	4.09 × 10^−1^	6.92 × 10^−1^	6.04 × 10^−1^	5.97 × 10^−1^	3.11 × 10^−1^	4.07 × 10^−1^	3.05 × 10^−1^	3.17 × 10^−1^	**1.00 × 10^−1^**
lena	2	Mean	14.9888	14.9666	14.9883	14.9503	**14.9957**	14.9672	14.9481	14.9729	14.9860	14.9910
		Std	4.00 × 10^−2^	5.50 × 10^−2^	4.76 × 10^−2^	7.25 × 10^−2^	**3.88 × 10^−2^**	5.27 × 10^−2^	8.02 × 10^−2^	4.41 × 10^−2^	3.90 × 10^−2^	3.89 × 10^−2^
	4	Mean	19.0903	19.0530	18.9173	18.9320	19.0882	18.9664	18.9328	19.0154	19.0863	**19.1352**
		Std	5.02 × 10^−2^	6.16 × 10^−2^	2.76 × 10^−1^	2.98 × 10^−1^	5.71 × 10^−2^	1.14 × 10^−1^	1.31 × 10^−1^	9.60 × 10^−2^	7.08 × 10^−2^	**2.94 × 10^−2^**
	6	Mean	21.6002	21.5676	21.0365	21.0846	21.3631	21.3089	21.2994	21.4763	21.5880	**21.8932**
		Std	2.75 × 10^−1^	2.92 × 10^−1^	5.29 × 10^−1^	5.58 × 10^−1^	4.43 × 10^−1^	3.07 × 10^−1^	3.18 × 10^−1^	2.24 × 10^−1^	1.78 × 10^−1^	**4.51 × 10^−2^**
	8	Mean	23.0934	22.9918	22.8177	22.8242	22.9642	22.9932	22.8443	23.0844	23.2527	**23.7823**
		Std	3.14 × 10^−1^	3.08 × 10^−1^	4.35 × 10^−1^	4.15 × 10^−1^	4.52 × 10^−1^	3.83 × 10^−1^	4.11 × 10^−1^	3.40 × 10^−1^	2.67 × 10^−1^	1.41 × 10^−1^
Average rank			4.45	5.04	6.87	7.00	5.87	6.65	7.09	5.25	4.57	**2.22**
Rank			2	4	8	9	6	7	10	5	3	**1**

**Table 9:** Mean and Std of all test images for FSIM in Otsu.

Image	Threshold	Metric	VPPSO	MELGWO	MEWOA	COA	DBO	GRO	CPO	ARO	POA	ACPOA
baboon	2	Mean	0.6871	0.6869	0.6864	0.6867	0.6871	0.6864	0.6863	0.6867	**0.6871**	**0.6871**
		Std	1.11 × 10^−4^	1.30 × 10^−3^	3.09 × 10^−3^	2.25 × 10^−3^	2.16 × 10^−4^	3.04 × 10^−3^	4.35 × 10^−3^	1.41 × 10^−3^	**0.00 × 10^0^**	**0.00 × 10^0^**
	4	Mean	0.8189	0.8200	0.8229	0.8142	0.8186	0.8169	0.8142	0.8188	0.8217	**0.8233**
		Std	3.86 × 10^−3^	4.61 × 10^−3^	1.08 × 10^−2^	1.31 × 10^−2^	7.39 × 10^−3^	1.30 × 10^−2^	1.54 × 10^−2^	9.51 × 10^−3^	4.46 × 10^−3^	**1.05 × 10^−3^**
	6	Mean	0.8842	0.8798	0.8833	0.8792	0.8703	0.8799	0.8762	0.8824	0.8859	**0.8861**
		Std	1.22 × 10^−2^	1.45 × 10^−2^	2.33 × 10^−2^	2.28 × 10^−2^	1.88 × 10^−2^	1.61 × 10^−2^	2.08 × 10^−2^	1.29 × 10^−2^	1.11 × 10^−2^	**2.54 × 10^−3^**
	8	Mean	0.9113	0.9121	0.9051	0.9027	0.9071	0.9101	0.9077	0.9150	0.9158	**0.9223**
		Std	1.66 × 10^−2^	1.65 × 10^−2^	1.73 × 10^−2^	2.16 × 10^−2^	1.65 × 10^−2^	1.82 × 10^−2^	1.76 × 10^−2^	1.27 × 10^−2^	1.65 × 10^−2^	**3.85 × 10^−3^**
bank	2	Mean	**0.7530**	0.7529	0.7529	0.7525	0.7529	0.7528	0.7529	0.7529	**0.7530**	**0.7530**
		Std	**1.13 × 10^−16^**	9.80 × 10^−4^	1.00 × 10^−3^	2.09 × 10^−3^	1.92 × 10^−4^	5.77 × 10^−4^	1.15 × 10^−3^	6.41 × 10^−4^	**1.13 × 10^−16^**	**1.13× 10^−16^**
	4	Mean	0.8344	0.8337	0.8340	0.8308	0.8342	0.8342	0.8320	0.8332	0.8343	**0.8352**
		Std	1.62 × 10^−3^	2.75 × 10^−3^	8.66 × 10^−3^	7.64 × 10^−3^	3.55 × 10^−3^	4.77 × 10^−3^	4.82 × 10^−3^	4.02 × 10^−3^	2.11 × 10^−3^	**4.20 × 10^−4^**
	6	Mean	0.8785	0.8760	0.8717	0.8738	0.8770	0.8761	0.8736	0.8749	0.8775	**0.8816**
		Std	3.60 × 10^−3^	6.54 × 10^−3^	8.52 × 10^−3^	7.52 × 10^−3^	5.84 × 10^−3^	5.17 × 10^−3^	5.85 × 10^−3^	4.77 × 10^−3^	3.29 × 10^−3^	**4.36 × 10^−4^**
	8	Mean	0.9017	0.9011	0.8971	0.8957	0.8989	0.8994	0.8977	0.9024	0.9035	**0.9089**
		Std	5.38 × 10^−3^	7.32 × 10^−3^	9.08 × 10^−3^	8.24 × 10^−3^	6.83 × 10^−3^	5.65 × 10^−3^	6.74 × 10^−3^	5.00 × 10^−3^	3.96 × 10^−3^	**2.20 × 10^−3^**
camera	2	Mean	0.7662	**0.7663**	0.7661	0.7661	0.7662	0.7662	0.7656	0.7660	0.7661	0.7661
		Std	5.42 × 10^−4^	4.69 × 10^−4^	2.85 × 10^−4^	8.36 × 10^−4^	2.14 × 10^−4^	7.33 × 10^−4^	1.60 × 10^−3^	1.09 × 10^−3^	**0.00 × 10^0^**	**0.00 × 10^0^**
	4	Mean	**0.8342**	0.8332	0.8290	0.8307	0.8327	0.8280	0.8297	0.8317	0.8302	0.8326
		Std	5.69 × 10^−3^	8.65 × 10^−3^	9.18 × 10^−3^	9.55 × 10^−3^	7.27 × 10^−3^	9.30 × 10^−3^	8.87 × 10^−3^	8.28 × 10^−3^	6.16 × 10^−3^	**8.65 × 10^−4^**
	6	Mean	0.8697	0.8654	0.8629	0.8628	0.8667	0.8625	0.8663	0.8677	0.8718	**0.8781**
		Std	8.02 × 10^−3^	1.24 × 10^−2^	1.12 × 10^−2^	9.41 × 10^−3^	1.00 × 10^−2^	1.19 × 10^−2^	1.13 × 10^−2^	9.31 × 10^−3^	7.72 × 10^−3^	**1.12 × 10^−3^**
	8	Mean	0.8932	0.8929	0.8844	0.8835	0.8879	0.8898	0.8879	0.8906	0.8920	**0.9016**
		Std	8.31 × 10^−3^	1.02 × 10^−2^	1.51 × 10^−2^	1.05 × 10^−2^	1.02 × 10^−2^	9.53 × 10^−3^	1.02 × 10^−2^	1.10 × 10^−2^	8.85 × 10^−3^	**2.47 × 10^−3^**
face	2	Mean	0.6045	0.6048	**0.6049**	0.6049	0.6047	0.6043	0.6042	0.6044	0.6048	0.6049
		Std	7.08 × 10^−4^	9.85 × 10^−4^	4.26 × 10^−4^	9.39 × 10^−4^	6.80 × 10^−4^	9.15 × 10^−4^	1.49 × 10^−3^	1.12 × 10^−3^	1.74 × 10^−4^	**2.26× 10^−16^**
	4	Mean	0.7532	0.7518	0.7486	0.7510	0.7536	0.7490	0.7471	0.7517	0.7518	**0.7542**
		Std	2.32 × 10^−3^	4.77 × 10^−3^	6.66 × 10^−3^	7.39 × 10^−3^	3.88 × 10^−3^	7.27 × 10^−3^	8.49 × 10^−3^	5.73 × 10^−3^	5.02 × 10^−3^	**8.56 × 10^−4^**
	6	Mean	0.8384	0.8339	0.8181	0.8225	0.8273	0.8240	0.8214	0.8283	0.8358	**0.8435**
		Std	6.14 × 10^−3^	1.02 × 10^−2^	1.23 × 10^−2^	1.40 × 10^−2^	1.47 × 10^−2^	9.81 × 10^−3^	9.43 × 10^−3^	8.92 × 10^−3^	5.58 × 10^−3^	**1.40 × 10^−3^**
	8	Mean	0.8819	0.8738	0.8552	0.8669	0.8655	0.8613	0.8645	0.8726	0.8779	**0.8950**
		Std	7.98 × 10^−3^	9.89 × 10^−3^	1.76 × 10^−2^	1.46 × 10^−2^	1.51 × 10^−2^	9.46 × 10^−3^	1.11 × 10^−2^	9.08 × 10^−3^	7.67 × 10^−3^	**1.53 × 10^−3^**
lena	2	Mean	0.6711	0.6709	0.6710	0.6692	0.6707	0.6707	0.6690	0.6703	0.6713	**0.6713**
		Std	1.23 × 10^−3^	1.33 × 10^−3^	1.52 × 10^−3^	4.85 × 10^−3^	1.90 × 10^−3^	1.57 × 10^−3^	3.50 × 10^−3^	2.52 × 10^−3^	1.09 × 10^−3^	**1.05 × 10^−3^**
	4	Mean	0.7810	0.7793	0.7752	0.7768	0.7806	0.7791	0.7782	0.7789	**0.7810**	0.7797
		Std	1.91 × 10^−3^	3.09 × 10^−3^	8.27 × 10^−3^	9.51 × 10^−3^	1.80 × 10^−3^	4.11 × 10^−3^	5.29 × 10^−3^	3.54 × 10^−3^	2.36 × 10^−3^	**6.78 × 10^−4^**
	6	Mean	0.8414	0.8412	0.8319	0.8336	0.8358	0.8354	0.8340	0.8398	0.8439	**0.8510**
		Std	9.05 × 10^−3^	7.91 × 10^−3^	1.46 × 10^−2^	1.38 × 10^−2^	1.14 × 10^−2^	8.81 × 10^−3^	1.03 × 10^−2^	8.12 × 10^−3^	7.25 × 10^−3^	**1.82 × 10^−3^**
	8	Mean	0.8706	0.8685	0.8657	0.8647	0.8681	0.8681	0.8652	0.8694	0.8741	**0.8840**
		Std	6.79 × 10^−3^	9.28 × 10^−3^	1.04 × 10^−2^	9.02 × 10^−3^	1.15 × 10^−2^	1.07 × 10^−2^	8.96 × 10^−3^	8.30 × 10^−3^	6.64 × 10^−3^	**2.60 × 10^−3^**
Average rank			4.58	5.29	6.54	6.65	5.86	6.08	6.79	5.34	4.70	**3.17**
Rank			2	4	8	9	6	7	10	5	3	**1**

**Table 10:** Mean and Std of all test images for SSIM in Otsu.

Image	Threshold	Metric	VPPSO	MELGWO	MEWOA	COA	DBO	GRO	CPO	ARO	POA	ACPOA
baboon	2	Mean	0.7202	**0.7256**	**0.7277**	0.4679	0.4686	0.4674	0.4666	0.4689	0.4688	0.4688
		Std	7.92 × 10^−3^	8.22 × 10^−3^	2.06 × 10^−2^	4.14 × 10^−3^	1.01 × 10^−3^	3.48 × 10^−3^	5.66 × 10^−3^	2.88 × 10^−3^	2.85 × 10^−16^	**2.82 × 10^−16^**
	4	Mean	0.7213	0.7171	**0.7271**	0.7136	0.7201	0.7185	0.7097	0.7231	0.7261	0.7239
		Std	1.27 × 10^−2^	1.39 × 10^−2^	1.15 × 10^−2^	2.49 × 10^−2^	1.51 × 10^−2^	2.45 × 10^−2^	2.86 × 10^−2^	1.66 × 10^−2^	8.40 × 10^−3^	**1.63 × 10^−3^**
	6	Mean	0.8291	0.8238	0.8276	0.8197	0.8107	0.8236	0.8164	0.8268	0.8328	**0.8352**
		Std	1.73 × 10^−2^	2.12 × 10^−2^	3.55 × 10^−2^	3.49 × 10^−2^	2.99 × 10^−2^	2.35 × 10^−2^	3.22 × 10^−2^	1.85 × 10^−2^	1.68 × 10^−2^	**4.33 × 10^−3^**
	8	Mean	0.8727	0.8756	0.8648	0.8596	0.8662	0.8692	0.8683	0.8786	0.8794	**0.8917**
		Std	2.25 × 10^−2^	2.05 × 10^−2^	2.32 × 10^−2^	3.08 × 10^−2^	2.20 × 10^−2^	2.37 × 10^−2^	2.28 × 10^−2^	1.53 × 10^−2^	2.03 × 10^−2^	**4.40 × 10^−3^**
bank	2	Mean	0.6361	0.6362	**0.6366**	0.6352	0.6363	0.6361	0.6356	0.6363	0.6364	0.6364
		Std	4.52 × 10^−4^	5.94 × 10^−4^	1.29 × 10^−3^	4.17 × 10^−3^	2.14 × 10^−4^	1.38 × 10^−3^	2.56 × 10^−3^	1.06 × 10^−3^	**0.00 × 10^0^**	**0.00 × 10^0^**
	4	Mean	0.7428	0.7415	0.7441	0.7402	0.7433	**0.7462**	0.7421	0.7422	0.7434	0.7453
		Std	3.58 × 10^−3^	5.22 × 10^−3^	1.27 × 10^−2^	1.22 × 10^−2^	6.60 × 10^−3^	8.64 × 10^−3^	8.81 × 10^−3^	7.24 × 10^−3^	3.65 × 10^−3^	**4.46 × 10^−4^**
	6	Mean	0.8105	0.8074	0.8040	0.8066	0.8091	0.8089	0.8041	0.8044	0.8103	**0.8152**
		Std	8.60 × 10^−3^	1.06 × 10^−2^	1.37 × 10^−2^	1.41 × 10^−2^	1.15 × 10^−2^	1.13 × 10^−2^	1.25 × 10^−2^	9.54 × 10^−3^	7.16 × 10^−3^	**1.44 × 10^−3^**
	8	Mean	0.8436	0.8457	0.8413	0.8368	0.8432	0.8438	0.8404	0.8467	0.8493	**0.8556**
		Std	9.99 × 10^−3^	1.05 × 10^−2^	1.46 × 10^−2^	1.14 × 10^−2^	1.07 × 10^−2^	1.00 × 10^−2^	1.29 × 10^−2^	7.47 × 10^−3^	5.74 × 10^−3^	**3.02 × 10^−3^**
camera	2	Mean	0.6364	0.6363	0.6359	0.6195	0.6203	0.6198	0.6197	0.6198	0.6204	**0.6404**
		Std	1.13× 10^−16^	1.06 × 10^−3^	1.82 × 10^−3^	2.83 × 10^−3^	3.32 × 10^−4^	6.50 × 10^−4^	1.01 × 10^−3^	7.13 × 10^−4^	1.13× 10^−16^	**0.00 × 10^0^**
	4	Mean	0.7137	0.6917	0.6947	0.6995	0.6948	0.7056	0.6965	0.7087	0.7445	**0.7583**
		Std	3.60 × 10^−2^	2.74 × 10^−2^	4.70 × 10^−2^	4.17 × 10^−2^	3.87 × 10^−2^	3.54 × 10^−2^	4.65 × 10^−2^	3.85 × 10^−2^	2.30 × 10^−2^	**2.48 × 10^−3^**
	6	Mean	0.7803	0.7787	0.7641	0.7610	0.7826	0.7737	0.7784	0.7841	0.7948	**0.8036**
		Std	2.71 × 10^−2^	2.89 × 10^−2^	4.07 × 10^−2^	4.24 × 10^−2^	3.13 × 10^−2^	3.57 × 10^−2^	4.02 × 10^−2^	2.97 × 10^−2^	2.06 × 10^−2^	**2.41 × 10^−3^**
	8	Mean	0.8201	0.8181	0.8119	0.8131	0.8088	0.8182	0.8145	0.8193	0.8191	**0.8300**
		Std	1.81 × 10^−2^	2.42 × 10^−2^	3.55 × 10^−2^	3.09 × 10^−2^	3.26 × 10^−2^	2.52 × 10^−2^	3.27 × 10^−2^	3.08 × 10^−2^	2.39 × 10^−2^	**4.78 × 10^−3^**
face	2	Mean	0.5228	0.5231	0.5238	0.5236	0.5233	0.5226	0.5225	0.5227	0.5237	**0.5238**
		Std	1.56 × 10^−3^	2.42 × 10^−3^	9.58 × 10^−4^	2.58 × 10^−3^	1.44 × 10^−3^	2.19 × 10^−3^	3.49 × 10^−3^	2.40 × 10^−3^	4.43 × 10^−4^	**2.26 × 10^−16^**
	4	Mean	0.7050	0.7046	0.7006	0.7046	0.7071	0.7006	0.6984	0.7045	0.7031	**0.7074**
		Std	4.39 × 10^−3^	9.13 × 10^−3^	9.47 × 10^−3^	1.11 × 10^−2^	8.23 × 10^−3^	1.36 × 10^−2^	1.62 × 10^−2^	1.10 × 10^−2^	9.48 × 10^−3^	**1.59 × 10^−3^**
	6	Mean	0.7952	0.7908	0.7763	0.7816	0.7859	0.7810	0.7800	0.7842	0.7923	**0.7999**
		Std	6.61 × 10^−3^	1.12 × 10^−2^	1.32 × 10^−2^	1.68 × 10^−2^	1.42 × 10^−2^	1.22 × 10^−2^	1.30 × 10^−2^	1.23 × 10^−2^	6.52 × 10^−3^	**1.91 × 10^−3^**
	8	Mean	0.8449	0.8386	0.8172	0.8307	0.8294	0.8236	0.8272	0.8359	0.8416	**0.8576**
		Std	8.22 × 10^−3^	1.08 × 10^−2^	1.93 × 10^−2^	1.55 × 10^−2^	1.60 × 10^−2^	1.09 × 10^−2^	1.47 × 10^−2^	1.13 × 10^−2^	9.32 × 10^−3^	**2.57 × 10^−3^**
lena	2	Mean	0.5452	0.5443	0.5449	0.5412	0.5447	0.5443	0.5406	0.5434	0.5455	**0.5456**
		Std	2.58 × 10^−3^	3.19 × 10^−3^	3.30 × 10^−3^	8.93 × 10^−3^	3.45 × 10^−3^	2.96 × 10^−3^	6.38 × 10^−3^	4.58 × 10^−3^	2.24 × 10^−3^	**2.17 × 10^−3^**
	4	Mean	**0.6756**	0.6745	0.6727	0.6725	0.6752	0.6741	0.6744	0.6737	0.6754	0.6756
		Std	1.39 × 10^−3^	3.63 × 10^−3^	8.96 × 10^−3^	1.07 × 10^−2^	2.25 × 10^−3^	4.73 × 10^−3^	6.48 × 10^−3^	4.47 × 10^−3^	1.62 × 10^−3^	**7.13 × 10^−4^**
	6	Mean	0.7468	0.7484	0.7409	0.7414	0.7426	0.7462	0.7398	0.7500	0.7535	**0.7596**
		Std	1.23 × 10^−2^	1.25 × 10^−2^	2.29 × 10^−2^	2.19 × 10^−2^	1.75 × 10^−2^	1.56 × 10^−2^	1.49 × 10^−2^	1.24 × 10^−2^	1.00 × 10^−2^	**3.15 × 10^−3^**
	8	Mean	0.7860	0.7898	0.7914	0.7843	0.7871	0.7911	0.7896	0.7958	0.7936	**0.8103**
		Std	1.24 × 10^−2^	1.82 × 10^−2^	2.29 × 10^−2^	1.76 × 10^−2^	2.06 × 10^−2^	1.98 × 10^−2^	2.17 × 10^−2^	1.91 × 10^−2^	1.48 × 10^−2^	8.58 × 10^−3^
Average rank			5.15	5.21	5.97	6.40	5.63	6.07	6.72	5.41	5.07	**3.39**
Rank			3	4	7	9	6	8	10	5	2	**1**

## Data Availability

All data in this paper are included in the manuscript.
